# Network Theory Analysis of Antibody-Antigen Reactivity Data: The Immune Trees at Birth and Adulthood

**DOI:** 10.1371/journal.pone.0017445

**Published:** 2011-03-08

**Authors:** Asaf Madi, Dror Y. Kenett, Sharron Bransburg-Zabary, Yifat Merbl, Francisco J. Quintana, Alfred I. Tauber, Irun R. Cohen, Eshel Ben-Jacob

**Affiliations:** 1 School of Physics and Astronomy, Tel Aviv University, Tel Aviv, Israel; 2 Faculty of Medicine, Tel Aviv University, Tel Aviv, Israel; 3 Department of Immunology, Weizmann Institute of Science, Rehovot, Israel; 4 Department of Systems Biology, Harvard Medical School, Boston, Massachusetts, United States of America; 5 Center for Neurologic Diseases, Brigham and Women's Hospital, Harvard Medical School, Boston, Massachusetts, United States of America; 6 School of Medicine, Boston University, Boston, Massachusetts, United States of America; 7 Center for Theoretical and Biological Physics, University of California San Diego, La Jolla, California, United States of America; City of Hope National Medical Center and Beckman Research Institute, United States of America

## Abstract

**Motivation:**

New antigen microarray technology enables parallel recording of antibody reactivities with hundreds of antigens. Such data affords system level analysis of the immune system's organization using methods and approaches from network theory. Here we measured the reactivity of 290 antigens (for both the IgG and IgM isotypes) of 10 healthy mothers and their term newborns. We constructed antigen correlation networks (or immune networks) whose nodes are the antigens and the edges are the antigen-antigen reactivity correlations, and we also computed their corresponding minimum spanning trees (MST) – maximal information reduced sub-graphs. We quantify the network organization (topology) in terms of the network theory divergence rate measure and rank the antigen importance in the full antigen correlation networks by the eigen-value centrality measure. This analysis makes possible the characterization and comparison of the IgG and IgM immune networks at birth (newborns) and adulthood (mothers) in terms of topology and node importance.

**Results:**

Comparison of the immune network topology at birth and adulthood revealed partial conservation of the IgG immune network topology, and significant reorganization of the IgM immune networks. Inspection of the antigen importance revealed some dominant (in terms of high centrality) antigens in the IgG and IgM networks at birth, which retain their importance at adulthood.

## Introduction

The recently introduced new antigen microarray chip enables detection in parallel of the patterns of antibodies binding to hundreds of antigens, and so provides a system-level view of the antibody repertoire [1], [2], [3]. Recently, we analyzed autoantibody reactivity data of IgM and IgG isotypes present in the sera of 10 healthy mothers at childbirth and in the sera of the cord bloods of their offspring. The data were obtained using an antigen chip with 290 antigens (see Supporting Information S1). The antigen-antigen correlation matrices revealed that the IgG repertoires of each mother and her offspring were very closely related and distinct for each mother-newborn pair [4]. The IgM repertoires, in contrast, differed markedly between mothers and offspring; each mother manifested a different pattern of IgM reactivities that was distinct from her offspring's cord IgM repertoire. However, the IgM reactivities of each of the newborn samples manifested very similar antigen-binding profiles indicating that in utero each developing fetus produced autoantibodies to a similar set of self-molecules. A subsequent analysis of the data revealed that the reactivity profiles to certain self-molecules were highly correlated as sets of functional antigen-reactivity cliques [4].

Here, we extended the study of these data by applying graph and network theory analysis methods [5], [6], [7]; the aim was to confirm the previous findings and search for additional insights into the internal structures of the natural autoantibody repertoires. To this end, we present the antigen-antigen correlation matrices in terms of immune correlation networks (or immune networks). Each node in these networks represents a specific antigen and the edges that connect the nodes represent the corresponding antigen-antigen correlations. To extract the most relevant information, we evaluated the corresponding Minimum Spanning Trees (MST), or immune trees, for the networks of antigen correlations computed from the correlation matrices [5], [6], [7], [8]. The MST is a widely used sub-graph of the complete network that is constructed using a special algorithm that enables to extract the most relevant information from the full network [9]. In the complete network, every node is linked to all other nodes with most of the links representing very weak correlations. Therefore, the complete graph contains a large amount of non-significant information that could mask the essential motifs. The objective of the MST algorithm is to select the subset of more informative links (regarding the hierarchical structure of the system) and reduce the complete all-to-all network (that contains *N*(*N*-1) links) to a representative sub-graph (that contains only *N*-1 links). Hence, generating the maximum information immune networks (or immune trees) by the MST, makes it possible to investigate the essential organization motifs, such as the network topological organization.

We assessed and compared the topological organization of the IgG and IgM immune trees of the mothers and newborns. Next, the networks of the two subject groups, mothers and newborns, were compared by employing the widely used divergence rate measure [10]. The analysis revealed high topological similarity between the newborns' and mothers' IgG networks and significant topological differences between the newborns' and the mothers' IgM networks. These results indicate partial conservation of the IgG immune network topology at birth and adulthood, and significant reorganization of the IgM immune networks during the immune system development. This observation is consistent with the fact that most of the IgG antibodies in cord blood originate from the mother, as they are actively transported across the placenta to the developing fetus [11]. In contrast, the IgM antibodies do not cross the placenta, so IgM autoantibodies in cord blood are necessarily produced by the developing fetus during pregnancy before birth [4], [12].

Much effort has been devoted to assessing the importance of nodes in complex biological networks such as gene transcriptional regulatory networks, protein interaction networks and neural networks. The commonly used measures of node importance include node degree, node centrality, betweenness, and node vulnerability score [13]. Here, we ranked the antigen importance in the complete correlation networks using the eigen-value centrality measure [6], [14]. This measure assigns a high score to the nodes that are strongly linked (have high correlations) with high-score nodes. The node centrality retains information that could be lost by the construction of the MST reduced graph. So, we developed a hybrid presentation in which the node centrality information is superimposed on the evaluated MST. This is simply done by coloring each node according to its eigen-value centrality score. The results presented here indicate that, indeed, the hybrid analysis revealed additional features beyond those that could be obtained by each analysis (MST and node centrality) alone.

## Methods

### Serum samples

Blood samples were obtained by random availability from 10 healthy women at the onset of term labor and from 10 serum samples of the cord blood of their newborns, in the course of normal procedures. All samples were collected with informed consent and approval by the Institutional Review Board (Helsinki Committee) of the Tel-Aviv Sourasky Medical Center. The newborns were healthy at the term of pregnancy (weeks 38–42) and normal in development and weight for gestational age. The blood samples were allowed to clot at room temperature. After centrifugation, sera were collected and stored at −20°C [4], [12].

### Antigens

305 antigens were spotted on each microarray, as described previously [4], [12]. For the most part, we used the same antigens as in the previous studies of natural autoimmune repertoires [4], [12]; these included proteins, synthetic peptides from the sequences of key proteins, nucleotides, phospholipids, and other self and non-self molecules. See Supporting Information S1 for the full list.

### Antigen microarray

Antigen microarrays were prepared and studied as described previously [1], [2], [3].

### Data preprocessing and background filtering

Antigen reactivity was defined by the mean intensity of the 4 replicates binding to that antigen on the microarray; however, antigen intensities with mean value lower than 1000 in removed from the datasets leaving us with 290 antigens. Each chip was then normalized by its mean reactivity divided by the standard deviation. This was done in order to account for differences in total protein concentrations that affect the background intensity level [4], [12].

### The correlation matrices and their collective normalization

Following Madi *et al.* [4], we started by calculating the antigen correlation matrices from the antibody reactivity data obtained using the antigen microarray technology. The correlations between the antigen reactivity profiles (the reactivities of the antigen in all subjects), were calculated by Pearson's formula [15]:
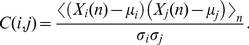
(1)


Where 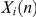 and 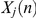 are the reactivity of antigens *i* and *j* of subject *n* and 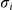 and 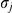 are the STD of the reactivity profiles of antigens *i* and *j*. Note that the antigen-antigen correlations (or for simplicity the antigen correlations) for all pairs of antigen define a symmetric correlation matrix whose 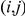 element is the correlation between antigens *i* and *j*.

Similar to neural and gene networks, the immune system can exhibit activated and inhibited reactivity responses; both positive and negative antigen correlations contain important information. To ease the calculations while retaining the information about negative correlations, we start by transforming the Pearson correlation values 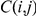 from the originally computed range of (−1,+1) to the range of (0,1). We saved the original values of the correlations in the range (−1,+1) and used these values in the visualization of the edges in immune networks as is described in greater detail below.

Following Baruchi et al. [16], we normalized the correlation matrices using the following meta-correlations procedure: the meta-correlation 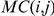 - the Pearson correlation between rows *i* and *j* of the correlation matrix after reordering. In the reordering process, the elements 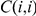 and 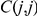 are removed from the calculation. The correlation vector for *i* is {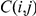, 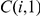, 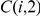,. . .}, and for *j* it is {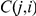, 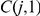, 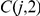,. . .}. In other words, the meta-correlation is a measure of the similarity between the correlations of antigen *i* with all other antigens and the correlations of antigen *j* with all other antigens. To ease the following calculations the meta-correlation matrix *MC* was transformed to the range of (0,1). The meta-correlations were then used to generate the normalized correlations 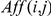 between antigens *i* and *j*, given by [4], [17], [18],
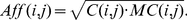
(2)


**Figure 1:**
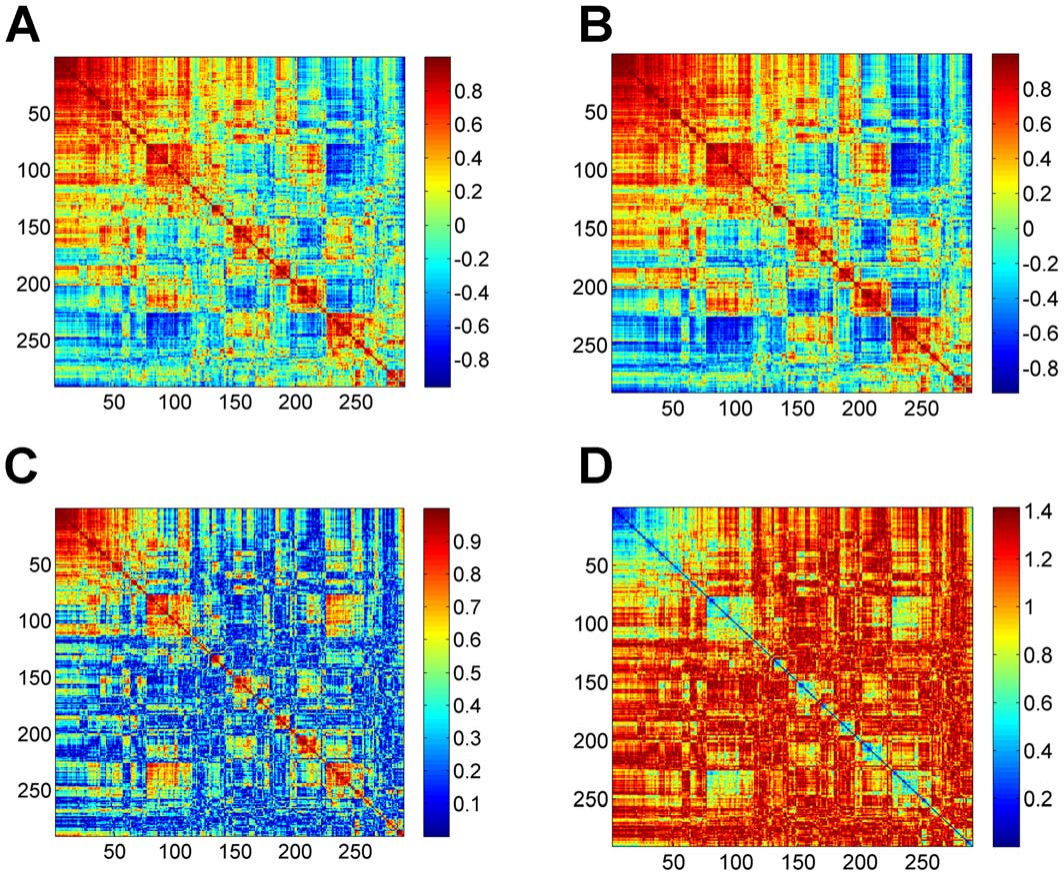
The process of transforming antigen-reactivity correlations into correlation-based distance: (A) correlation matrix; (B) normalized correlation matrix; (C) absolute value of the normalized correlation matrix after transformation to the range of (−1,1); and (D) distance matrix. The matrices presented here are for the mothers' IgM dataset. We note that for visualization proposes, the original correlation matrix (A) was reordered using the dendrogram algorithm, while all other matrices were reordered according to it.

The collective normalization further signifies features and can reveal collective motifs related to functional connectivity in the network [4], [17]. Additional comparison to the information embedded in the normalized correlation matrices using calculation of eigen-value entropies [19] can be found in Supporting Information S2. To retrieve the significant negative correlations, we transformed the normalized correlation to the range of (−1,1) and took the absolute values of the results using 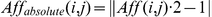. This process amplifies groups within the data set as is illustrated in Figure 1.

### Network representation of the correlation

In the current work we represented the correlation matrices by employing network theory approaches [5], [6], [7], [8]. In these immune network representations, each node corresponds to a specific antigen of the 290 antigens on the chip and the edges represent the antigen-antigen correlations (or normalized correlations) for a certain group of subjects (mothers or newborns).

### The weighted adjacency matrix

In network theory, an adjacency matrix commonly describes the network topology by containing the information of whether a link between two nodes exists or not. In the case of activity networks (e.g. correlations between stocks in financial networks [5] or synchronization between neuron activity patterns [14]), a weighted adjacency matrix, in which the "distances" between the nodes represent the activity similarity, is constructed. Here we used the ultrametric distance, suggested by Mantegna et al. [5], by transforming the normalized correlation between two nodes, 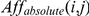, into a distance by
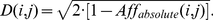
(3)


Defined this way, the distance 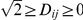 satisfies the metric requirements: 1.) 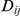 = 0 if and only if *i* = *j*, 2.) 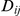 = 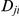, 3.) 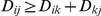, and also the requirements for ultrametricity [5].

In correlation based networks, such as the one described here, a weight, which is monotonically related to the correlation coefficient of each pair of elements, can be associated with each link. Therefore one can directly associate a weighted complete graph with the correlation matrix among N elements of interest.

### The reduced adjacency matrix and eigen-value centrality

Another approach to extract relevant information is by reduction of the weighted adjacency matrix into a reduced binary matrix, the elements of which are assigned values 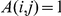, if the nodes *i* and *j* have a distance shorter than a threshold level, and 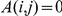. In simple words, this adjacency matrix describes a network in which two nodes *i* and *j* are linked if they are strongly correlated - the correlation between them is above a threshold level. Determining which threshold to use is not a simple task. Here we have chosen to use a measure of normalized STD of the principle eigenvector.

We begin by choosing a correlation threshold, and use it to create an adjacency matrix *A* as described above. We diagonalize the adjacency matrix, and focus on the principle eigenvector (the eigenvector corresponding to the largest eigenvalue). The centrality of each variable is defined as the weight of each variable in the principle eigenvector. We then compute the STD of the components for the principle eigenvector, and normalize it by the number of variables with a non-zero component. This allows us to search for a threshold that gives a compromise between high values of centrality and a small number of variables; in so doing, we can identify a significant sub-group of variables with the highest centrality in the network.

We then calculate the normalized STD of the eigenvalue centrality for different thresholds, ranging from 0 to 0.95 correlations. We test the normalized STD as a function of the correlation threshold for each of the datasets. The evaluation continues by investigating the second derivative of the resulting normalized STD's. This process revealed that the fluctuations in the second derivative of the STD begin at thresholds above 0.79 and more specifically above 0.82 for the maternal IgM, above 0.82 for the cord IgM, above 0.79 for the maternal IgG and above 0.86 for the cord IgG (see Supporting Information S3, Figures S1, S2 for additional explanations).

We used the information embedded in the *reduced adjacency matrix* described above to sort the antigens according to their reactivity dominance by their eigenvalue centrality score in the full correlation network. Mathematically, the eigenvector centrality 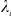 of node (i) is the largest eigen-value of the matrix equation
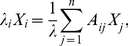
(4)where 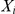 is the corresponding eigen-vector centrality of node *i* and *A* is the reduced adjacency matrix. According to the Perron–Frobenius theorem, 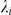 is positive [6].

### Minimum Spanning Tree

As was mentioned, a complete graph (in which each node is linked to all other nodes), contains too much irrelevant information (links that correspond to very weak correlations); hence, relevant information can be obscured [9]. The Minimum Spanning Tree (MST), is an algorithm designed to identify the informative links and reduce the complete network that contains 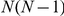 links to 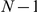 links.

Here we applied the commonly used Kruskal algorithm [20], [21] to compute the MST, but other algorithms can also be used [22], [23], [24]. This algorithm looks for a subset of the branches forming a tree that includes every node, where the total weight of all the branches in the tree, namely the score that is derived from the correlation, is minimized (see Supporting Information S4, Figure S3 for additional information)

### Network comparison based on divergence rate

We performed quantitative comparisons between the IgM and the IgG immune trees (Minimal Spanning Tree or MSTs) of the newborns and the mothers using the divergence rate measure developed by Lee *et al*. [10].

The divergence rate measure developed by Lee and Kim, 2006 [6] is based on the idea of quantification of the information difference between two process (variables) based on the notion of conditional entropy. In information theory, the specific conditional entropy 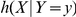 is the entropy of a process (variable), under the condition that another process *Y* is assigned the value y. The Conditional Entropy 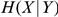 is then the average of 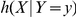 over all possible y that Y can take. It can be shown that 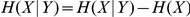, where 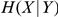 is the combined entropy of processes *X* and *Y* and 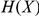 is the entropy of process *X*. The conditional entropy [6] has been used to define the metric distance or information distance 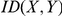 between two processes *X* and *Y* as 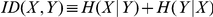.

Motivated by this idea, Lee and Kim [6], define the notion of the metric distance 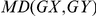 between two graphs 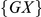 and 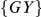 to be:
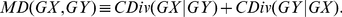
(5)


Where 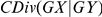 and 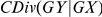 can be viewed as conditional divergences and are calculated as follows: First we define 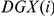 to be the sum of the topological distances from a node *i* to all its neighborhoods nodes 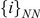. Then we define the conditional distances 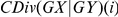 to be the sum of the topological distances in graph 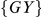 from node *i* to the group of nodes 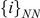 defined in graph 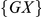. Note that these nodes, which are in the neighborhood of *i* in graph 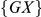 need not be in the neighborhood of *i* in the graph 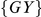. We also note that 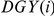 and 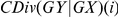 are defined in a similar way. With these definition at hand, 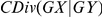 is defined to be:
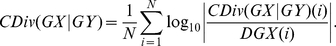
(6)


Note that in the informative sub-graphs studied here, each directed link from node *i* to node *j* corresponds to a topological distance 1 from *i* to *j* and we take the neighborhoods nodes 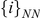 to be the nodes that have a topological distance 1 with node *i*. The topological distance between two nodes that are not directly connected by an edge is the number of directed edges of the shortest path connecting the two nodes.

## Results

We investigated the antigen correlation matrices of the IgG and IgM isotype antigen-reactivity data of 10 pairs of mothers and their newborns. We also studied the combined (or integrated) IgG and IgM correlation networks. The immune trees for the mothers and their newborns were calculated separately and compared.

### Global view of the combined antigen-reactivity networks

Antibodies of the IgM isotype are produced by B cells in the first phase of an antibody immune response, and IgM antibodies have been proposed to regulate the development of IgG autoantibodies [25] and prevent autoimmune diseases [26]. To test whether the IgM network might influence the IgG network, we analyzed the integrated correlation matrices of IgM and IgG datasets for the mothers and cords (see Supporting Information S5, Figure S4 for additional details).

**Figure 2:**
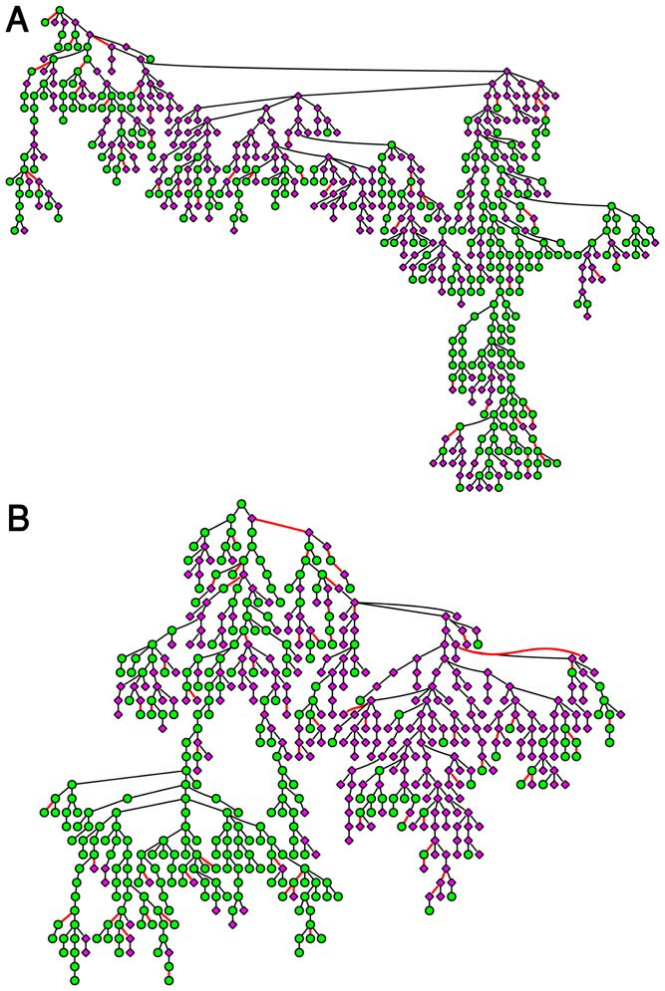
The IgM-IgG merged Minimal Spanning Trees. (A) The merged MST for the mothers. (B) The merged MST for the cords. The green (ellipse) and purple (diamond) nodes represent the IgG and IgM isotypes respectively. Negative correlations between two nodes are indicated by red lines.

Figure 2 shows the merged MST (immune trees) that correspond to the IgM-IgG integrated antibody correlation matrices. Inspection of these immune trees reveals a higher integration between the IgG and IgM isotypes for the mothers (Figure 2A) compared to the newborns (Figure 2B): for the mothers' tree, the two isotypes appear on the same branches, but tend to be segregated into different branches in the newborns' tree. To quantify these differences between the newborn and the maternal merged MSTs, we measured the topological distances between the different nodes in each of the trees, where the topological distance is measured in terms of the number of edges (see Supporting Information S6 for detailed statistics). The most significant result is that the average distance between IgG and IgM nodes in the newborns tree is 27.47 with a STD of 1.9 compared to 21.38 with STD of 1.6 in the maternal tree. This result suggests that natural maturation of the immune system from the newborn to young adulthood might lead to the evolution of greater coordination between antibody reactivities of the IgG and IgM isotypes.

### Immune tree architecture and node centrality

**Figure 3:**
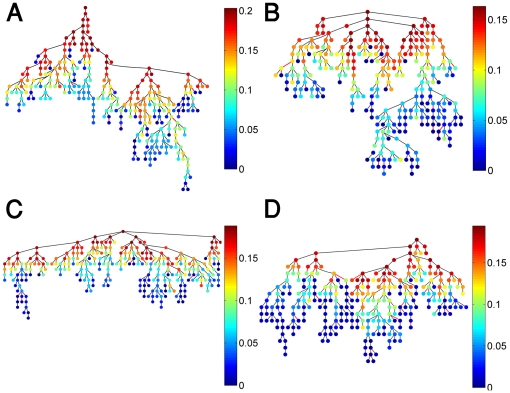
Hierarchical organization of the separated immune trees for the IgM and IgG isotypes. (A) The MST for of the maternal IgM; (B) The MST of the cords' IgM; (C) The MST of the maternal IgG; and (D) The MST of the cords' IgG. The nodes' colors indicate their centrality level from dark red for high centrality to dark blue for low centrality. The first node in the trees is the one with the highest centrality. Negative correlations between nodes are designated by red edges and constitute about 7–10% of the links.

Figure 3 shows the separated IgG and IgM MSTs for mothers and newborns (Figure 3A and 3B, respectively). The node colors indicated the eigen-value centrality, ranging from dark red for high centrality to dark blue for low centrality. In the construction of the trees, we selected the first node in each MST to be that with the highest centrality. Yet, we note that most of the nodes at the first levels of the trees (which are selected by the tree construction algorithm), also manifest a high centrality value. Negative correlations are indicated by red edges. It can be seen that the links within a single branch can turn from positive to negative, and vice versa. This finding indicates that both negative and positive relations between antigen-reactivities participate in connectivity throughout the MST network.

Quantified comparison between the MSTs (immune trees) revealed that the maternal (Figure 3C) and newborns (Figure 3D) IgG immune trees are very similar (with p-value = 0) followed by the similarity between the different isotypes within the groups (Figure 3B,D and Figure 3A,C). A moderate level of similarity was found between the newborns' IgG immune tree and the mothers' IgM immune tree and also between the newborns' IgM immune tree and the mothers' IgG immune tree. Significantly lower similarity was found between the IgM MSTs of the newborns and the mothers (Figure 3A,B p-value = 0). The similarity between the IgG immune trees of the mothers and newborns is consistent with the fact that IgG antibodies are actively transported from mother to her developing fetus [4], [11]. However, the difference between the IgM immune trees of the newborns and the mothers is a new observation, and indicates reorganization of the IgM network topology between birth and adulthood. We note that this result is consistent with our previous findings regarding the formation of antigen cliques in the maternal immune network [4].

### Central nodes

Analyses of node centrality (see Supporting Information S7, Figures S5, S6, S7, S8, S9 for details) revealed that about 10–15% of the nodes are highly ranked (or act as central nodes) - they manifest significantly higher centralities than those of the other nodes. These central nodes, or network hubs, are usually located at the first level of branches of the immune trees, as is seen in Figure 3.

We found that the central nodes of the IgG networks are prominently constituted by peptides of heat shock proteins (HSPs) (8 out of 10 for the mothers and 7 out of 10 for the cords) (see Table 1). In contrast, most of the central antigens of the IgM networks are associated with tissue and immune-related antigens (9 out of 10 for the mothers and 7 out of 10 for the cords) such as, cardiolipin, glucocerebroside and interleukin-4. It is conceivable that the dominance of HSP molecules as IgG hubs might be due to their over-representation on the antigen chip. However, the lack of highly ranked HSP molecules in the IgM network suggests that the dominance of these molecules in the IgG network is not an artifact. We also note that HSP60 appears to function as a biomarker of inflammation and stress for the immune system [27], [28], which suits the position of HSPs as hubs in the IgG networks.

**Table 1:** Antigen-reactivity hubs in IgM and IgG networks in maternal and newborns' sera.

Maternal IgM	Association	Newborns' IgM	Association	Maternal IgG	Association	Newborns' IgG	Association
GroEL-14	HSP	Vasoactive intestinal Peptide 16	Hormone	HSP70-37	HSP	HSP60-35	HSP
IFN-gamma	Immune	Poly aspartyl	Enzyme	Poly aspartyl		C peptide	Tissue
Kinetensin	Immune	Phospho-ethanolamine	Enzyme	GroEL-12	HSP	GroEL-24	HSP
Endothelin 2	Tissue	Matrix metalloproteinases protein	Protease	GroEL-29	HSP	HSP70-31	HSP
C peptide	Tissue	C peptide	Tissue	HSP70-36	HSP	somatostatin	Hormone
Spectrin	Tissue	GroEL-33	HSP	GroEL-4	HSP	Complement C9	Immune
Vasoactive intestinal peptide 16	Hormone	Interleukin 4	Immune	HSP60-28	HSP	GroEL-31	HSP
Cardiolipin	Tissue	Glucocerebroside	Tissue	HSP60-34	HSP	HSP60-23	HSP
Elastase	Enzyme	GroEL-10	HSP	Kinetensin	Immune	GroEL-6	HSP
Alpha 2 macroglubulin	Plasma protein	HSP60-21	HSP	GroEL-6	HSP	HSP70-39	HSP

### Centrality development

**Figure 4:**
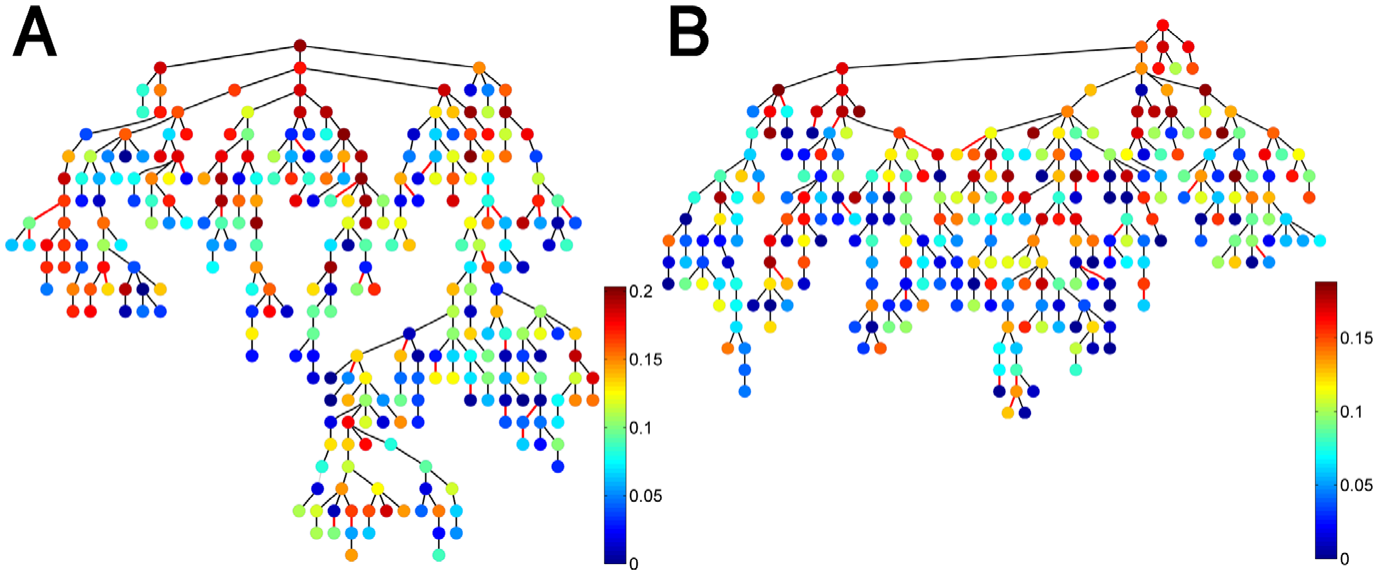
Centrality comparison between the immune networks of mothers and cords. In (A) and (B) we re-plot the cords' immune trees for the IgM and IgG shown in Figures 3B and 3D, while coloring the nodes according to the centrality calculated for the maternal IgM and IgG immune trees shown in Figures 3A and 3C.

To decipher the development of node centrality between birth and adulthood, we present in Figure 4 the newborns immune trees shown in Figures 3B and 3D, while coloring the nodes according to their centrality values as calculated for the maternal networks (Figures 3A and 3C). Doing so revealed that node centrality is partially retained between birth and adulthood both for the IgM and the IgG isotypes. This is mostly reflected by central nodes located at early levels of the corresponding immune trees. The result for IgG is expected, since it is consistent with the conservation of the network organization (similar topology of the newborns’ and mothers’ IgG immune trees). Regarding the IgM network (whose topology does change from birth to adulthood), the retained centrality suggest that there exist a core of central IgM reactivities shared by both newborns and mothers, despite the differences in the immune network overall architectures. These findings suggest that during the development of the immune system from birth to adulthood, some cliques of central antigens are conserved.

As mentioned above, the IgG isotypes are transferred during pregnancy from the mothers to the fetus. Hence, the findings of different IgG central nodes in the mothers and in the cords networks suggest that the IgG antibodies are transferred in a selective way. Such selective transfer can lead to the differences between the mothers and newborns IgG immune networks discovered here.

### The immune trees of the immune cliques

**Figure 5:**
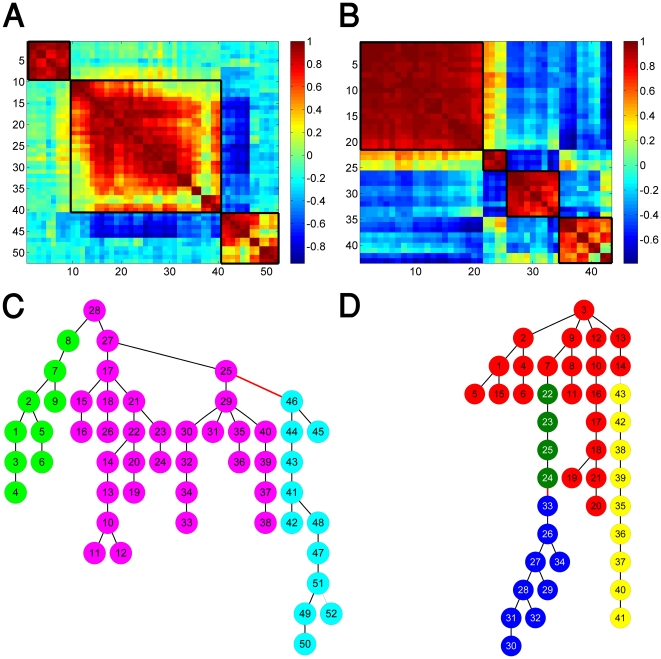
Immune trees of the immune cliques. The correlation matrices for the antigens that belong to the immune cliques identified in (Madi, et al., 2009), are shown in (A) for the maternal IgG isotypes and (B) for the maternal IgM isotypes. The corresponding immune trees for the maternal IgG cliques and the maternal IgM cliques are shown in (C) and (D), respectively. Note that the nodes colored green, purple and cyan in panel C correspond to the top left, middle and lower right clusters in panel A. The nodes colored red, green, blue and yellow in panel D correspond to the four clusters (from top left to bottom right) in panel B.

To test the consistency between the previously discovered antigen cliques (subgroups of highly correlated antigens) [4], and the immune tree organization detected here, we present in Figure 5 the immune trees for the immune cliques. This revealed additional information about the antigen cliques: the existence of negative relationships (marked by a red edge) and the relationships between cliques that are “mediated” by other cliques.

**Figure 6:**
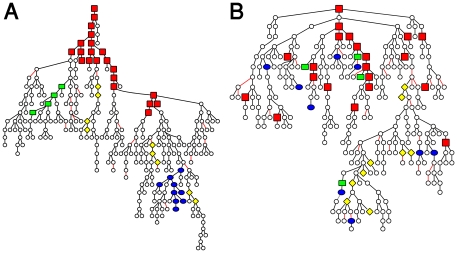
The clique association of the nodes on the immune trees. (A) The clique locations for the maternal IgM tree. The cliques association of the nodes is marked on the trees using different shapes and colors: clique 1 – red square, clique 2 – blue ellipse, clique 3 – dark green rectangle and clique 4 – yellow diamond. Note that although most of the cliques appear to be linked in the MST presentation, some were not linked, probably due to the loss of information in the dimension reduction process. (B) The clique locations on the cords' IgM tree. Panel B shows that maternal clique members are scattered in the network according to the cord dataset.

To decipher the functional relations of the immune cliques with other antigens we re-plotted in Figure 6A the maternal IgM immune tree (shown in Figure 3A), while coloring the nodes according to their immune-clique association. We found that most of the nodes that belong to the same clique are linked and that most of the central nodes belong to the strongest antigen clique – the clique with the highest antigen correlations (see Supporting Information S8 Figures S10, S11 for additional details).

In Figure 6B, we re-plotted the newborns' IgM immune tree (shown in Figure 3B), and marked the nodes according to their antigen clique associations in the maternal IgM network. This presentation provides a clear illustration of the different network organization of the newborns' IgM immune tree compared to the maternal IgM immune tree.

### Individual immune networks

**Figure 7:**
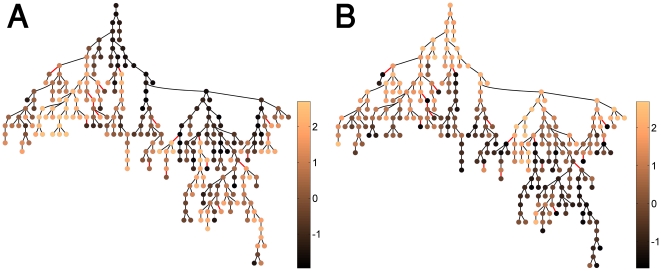
Individual immune trees. Hierarchical graph representations of two individual mother immune networks superimposed on the maternal IgM group dataset, as was presented in Figure 4A. The nodes are colored according to normalized antigen reactivity levels for two selected mothers, (A) and (B), from the most reactive node (light brown) to the least reactive nodes (dark brown). Note that the first nodes in each of the MSTs have high centrality value, as was shown previously in Figure 3.

The immune trees described above were derived from the reactivities of the groups of mothers and newborns. To compare the antigen-reactivity networks of individuals within a group, we superimposed on the maternal IgM immune tree (Figure 3A), the person-specific normalized IgM reactivity profiles (using a color code) of individual mothers. In Figure 7A and 7B, we show typical results for randomly selected two mothers. Similar results, of distinct differences between the individual immune trees, are obtained for other mothers as well. The results indicate that each mother has her own personal immune state reflected by the fact that each mother has her own person-specific reactivity profile to the 290 different antigens on the chip. Nevertheless, the existence of a well-defined group immune tree discloses a topological organization that is shared by the immune systems of the different mothers.

## Discussion

We constructed and analyzed the networks of autoantibody reactivities present in the blood sera of two groups of individuals – healthy mothers who had just given birth and their term newborn babies. This type of network analysis provides a powerful tool for simplifying complex systems, such as the immune system and for studying their components and their most informative interactions in order to identify their structure, topology and functions emerging from the organization of the collective of elements [29]. The present study is the first to describe the network- immune tree architecture of the natural autoantibody repertoires in healthy mothers and newborns. The analyses uncovered previously unrecognized features of natural autoantibodies in terms of network architecture and for the differences between mothers and cords:

1. Mothers and newborns repertoires manifest generally different network architectures. In the mothers' immune tree the IgG and IgM nodes are largely integrated, but remain distinct clusters in the newborns. The greater difference between IgM and IgG repertoires at birth could be explained by the fact that the congenital IgM and IgG repertoires may develop independently. After birth, the IgM and IgG repertoires together are influenced by the external antigenic environment. This result further suggests that natural maturation of the immune system from the newborn to young adulthood might lead to the evolution of greater coordination between antibody reactivities of the IgG and IgM isotypes. As was shown the networks are also characterized by negative relationships (correlations). It can be seen that the links within a single branch can turn from positive to negative, and vice versa. This finding indicates that both negative and positive relations between antigen reactivities participate in connectivity throughout the immune tree network.
2. Comparison of network topology between birth and adulthood reveals partial conservation of the IgG immune network topology between birth and adulthood and significant reorganization of the IgM immune networks. The similarity between the IgG immune trees of the mothers and newborns is expected as is explained above [4], [11]. However, the difference between the IgM immune trees of the newborns and the mothers is new and indicates reorganization of the IgM network topology between birth and adulthood. This result is consistent and provides additional support to our previous findings about the formation of antigen cliques in the maternal immune network [4].
3. The reactivities to central antigens in the mothers and newborns show both similarities and differences in both IgG and IgM isotypes. For example, antibodies to cytokines are central in the IgM trees of both mothers and newborns, but the maternal cytokine hub is INF-gamma, and the newborns' central cytokine is IL-4. Antibodies to heat shock proteins such as HSP60 are hubs in both maternal and newborns' IgM trees, but the HSP60 peptide epitopes differ in each tree (Table 1). Thus, the classes of antigens in central antibody reactivities can be conserved in molecular class, although they show differences in epitope selection. Moreover, in general we see that IgM reactivities to HSP molecules appear as the prominent central antigens in both mothers and newborns. We note, that heat shock proteins were initially discovered as participants in the cellular response to stress. It is now clear, however, that self and microbial HSPs also play an important role in the control of the immune response [30], [31]; in contrast, immune system molecules and tissue molecules are the prominent central antigens in the IgG immune trees of both mothers and cords.
4. Cliques of antigen reactivities, previously revealed by correlation analysis [4], are more tightly organized and integrated in the maternal network trees than they are in the newborns' network trees. However, this analysis further reveals additional information about the antigen cliques such as the existence of negative relationships and the relationships between cliques that are “mediated” by other cliques.

The results presented here illustrate the efficiency of the present method in revealing new and possibly important motifs of the immune system. For example the findings that show the persistence of central antigens from birth (newborns) to adulthood (mothers) might account for the reports that IgM repertoires show little change from early age [32], [33], [34]. These other studies, however, were done using crude tissue blots of undefined self-molecules; the defined-antigen microarray technology used here apparently made it possible for us to detect the changes in fine specificity of the autoimmune repertoire occurring subsequent to birth.

In general, the results presented here are consistent with the concept of the Immunological Homunculus, the idea that healthy immune repertoires contain certain T cells and B cells that have been positively selected to respond to key body molecules to form a functional “internal image” of the body [27], [28], [35], [36], [37], [38]. The internal image described here consists of natural autoantibodies interacting specifically with a small group of different extracellular, membrane, cytoplasmic, and nuclear self-antigens. The homunculus theory is based on the regularity of immune self-recognition consistently observed in healthy individuals. In practice, autoreactivity is not the aberration proposed by the Clonal-Selection Theory (CST) of adaptive immunity, but is actually structured within the functional architecture of the immune system. The hubs of self-reactivity we report here would seem to reflect the biases of selected self-recognition within groups of human populations [39]. Note that both the CST and the anti-idiotypic network paradigms [40], [41] are based on individual differences between the immune repertoires developed by individual subjects; the immunological homunculus idea, in contrast, highlights the existence of antigen reactivities shared by individuals within a population. The relative uniformity of IgM autoantibody repertoires in newborns as a group [4], [11] fits the homunculus idea; the demonstration of network MSTs with dominant hubs shown here provides an additional way to view the homunculus.

The analysis of immune system network architecture shown here and elsewhere [42] serve as an introduction to basic questions in systems immunology: What mechanisms connect nodes of antigen-reactivity, including anti-idiotypic networks of autoantibodies [43], [44]? What is the dynamic function of the relatively large number of central nodes that serve as hubs (∼10–15%)? And how is the architecture of the immune network tree modified by vaccinations, infections, neoplasia, autoimmune diseases, and other conditions that perturb immune homeostasis? The antigen microarray provides a tool to help study these questions. We are presently undertaking a longitudinal study of the evolution of the antibody repertoires of individual humans from birth with an array of antigens, including those directed to self-constituents and to foreign molecules.

## Supporting Information

Figure S1 **Normalized eigenvalue centrality STD, as function of correlation threshold.** Calculated for the maternal IgM (A), cords IgM (B), maternal IgG (C), and cords IgG (D).
(TIF)

Figure S2 **Second derivative of the normalized eigenvalue centrality STD, as function of correlation threshold.** Calculated for the maternal IgM (A), cords IgM (B), maternal IgG (C), and cords IgG (D). In all four cases, there is a significant change in normalized STD for thresholds larger than 0.79 and more specifically 0.79 for the cords' IgM, 0.85 for the cords' IgG, 0.89 for the maternal IgG and 0.85 for the maternal IgM.
(TIF)

Figure S3 **Illustration of the Kruskal algorithm.**
(TIF)

Figure S4 **The integrated correlation matrices of the IgM and IgG datasets.** Shown for (A) the mothers and (B) the cords. In each matrix, the IgM isotypes are in the top left frame and the IgG isotypes are in the bottom right frame. Both frames (isotypes) are ordered using a dendrogram algorithm demonstrating the relationships between correlated groups of antibodies of both isotypes.
(TIF)

Figure S5 **Antigen centrality values in descending order.** Calculated for the maternal IgM (A), cord IgM (B), maternal IgG (C), and cord IgG (D). Marked in red dots are the numbers of antigens whose centrality values constitute 30 percent of the total centrality values in the network. Note that for clearer visualization zero values were removed prior to plotting the data.
(TIF)

Figure S6 **Zipf plots of node centrality values.** Presented in descending order for the maternal IgM (A), cords' IgM (B), maternal IgG (C), and cords' IgG (D). Note that zero values were removed prior to plotting the data and the plots are presented in log scale.
(TIF)

Figure S7 **Semi-log plots of node centrality values.** Presented in descending order for the maternal IgM (A), cords IgM (B), maternal IgG (C), and cords IgG (D). Note that zero values were removed prior to plotting the data and the plots are presented in semi-log scale.
(TIF)

Figure S8 **Zipf plots of the descending sorted eigenvalues of the correlation matrices (absolute values).** Calculated for the maternal IgM (A), cords IgM (B), maternal IgG (C), and cords' IgG (D). Note that the plots are presented in log scale.
(TIF)

Figure S9 **Zipf plots of the descending sorted eigenvalues of the correlation matrices (absolute values).** Calculated for the maternal IgG and IgM (A), cords IgG and IgM (B). Note that the plots are presented in log scale.
(TIF)

Figure S10 **Robustness of the trees.** We calculated the distance from all nodes to all others and subtracted it from the original calculated distance (before removal of the random nodes), this process was repeated 100 times and the results were plotted for (A) the maternal IgG and (B) maternal IgM.
(TIF)

Figure S11 **Maternal conservation of FIGs.** For each of these randomly “trimmed” trees, we also calculated the average distance between all the nodes (members) within each FIG and subtracted it from the original calculated distance (before removal of the random nodes). (A) maternal IgM and (b) maternal IgG.
(TIF)

Supporting Information S1 **Complete list of antigens spotted on the antigen chip.**
(PDF)

Supporting Information S2 **The eigen-values' entropy of the correlation matrices.**
(DOC)

Supporting Information S3 **Calculation of the node centrality.**
(DOC)

Supporting Information S4 **Construction of the immune trees by the Kruskal algorithm.**
(DOC)

Supporting Information S5 **The integrated correlation matrices.**
(DOC)

Supporting Information S6 **The topological statistics of the immune trees.**
(DOC)

Supporting Information S7 **The central nodes – the network hubs.**
(DOC)

Supporting Information S8 **The immune tree robustness.**
(DOC)
