# Supplementary figures and images for: Network Theory Analysis of Antibody-Antigen Reactivity Data: The Immune Trees at Birth and Adulthood
Source: PLoS One. 2011 Mar 8;6(3):e17445. doi: 10.1371/journal.pone.0017445 (PMC3050881; doi:10.1371/journal.pone.0017445)

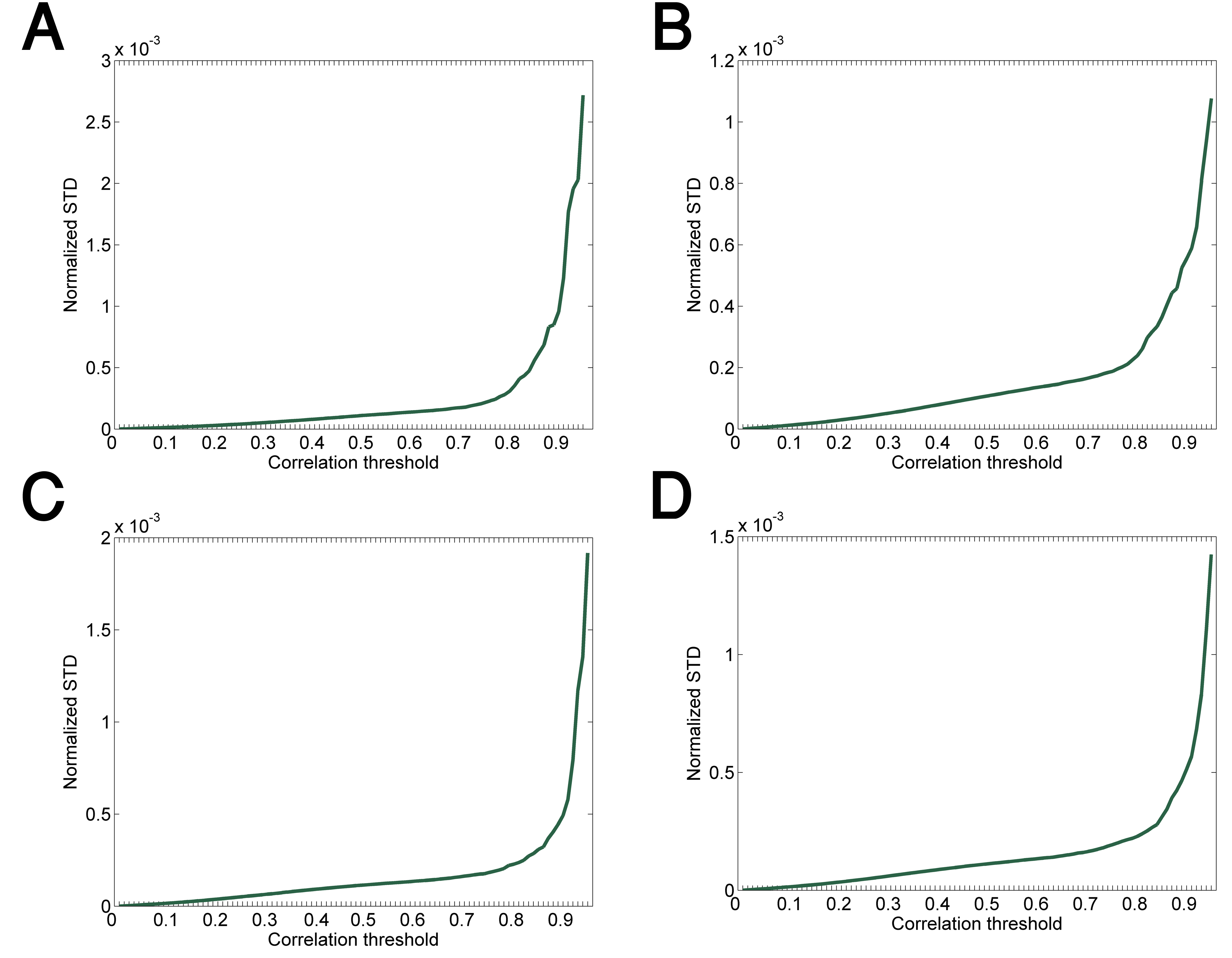

Supplement: Figure S1 — Normalized eigenvalue centrality STD, as function of correlation threshold. Calculated for the maternal IgM (A), cords IgM (B), maternal IgG (C), and cords IgG (D). (TIF) [file pone.0017445.s001.tif]

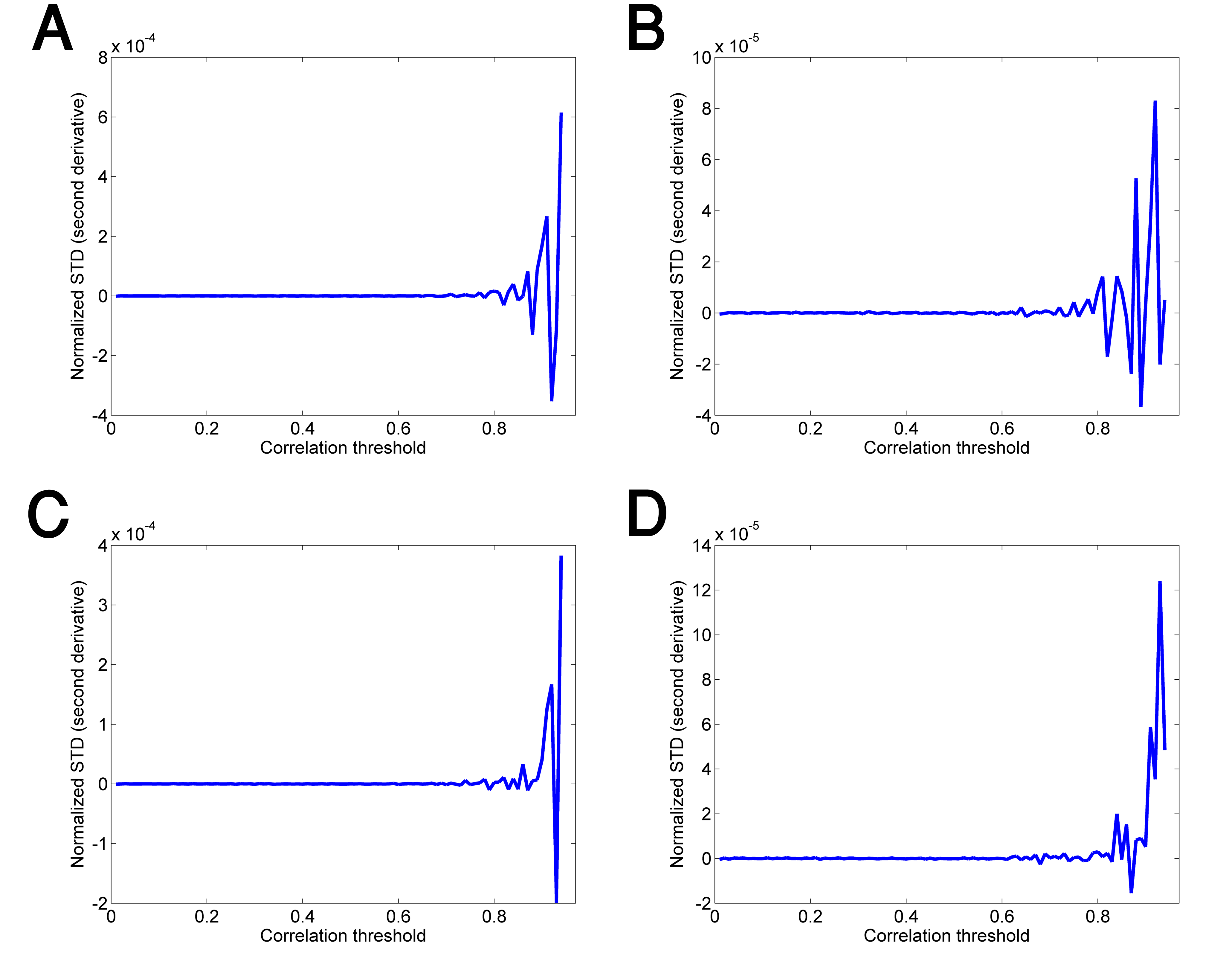

Supplement: Figure S2 — Second derivative of the normalized eigenvalue centrality STD, as function of correlation threshold. Calculated for the maternal IgM (A), cords IgM (B), maternal IgG (C), and cords IgG (D). In all four cases, there is a significant change in normalized STD for thresholds larger than 0.79 and more specifically 0.79 for the cords' IgM, 0.85 for the cords' IgG, 0.89 for the maternal IgG and 0.85 for the maternal IgM. (TIF) [file pone.0017445.s002.tif]

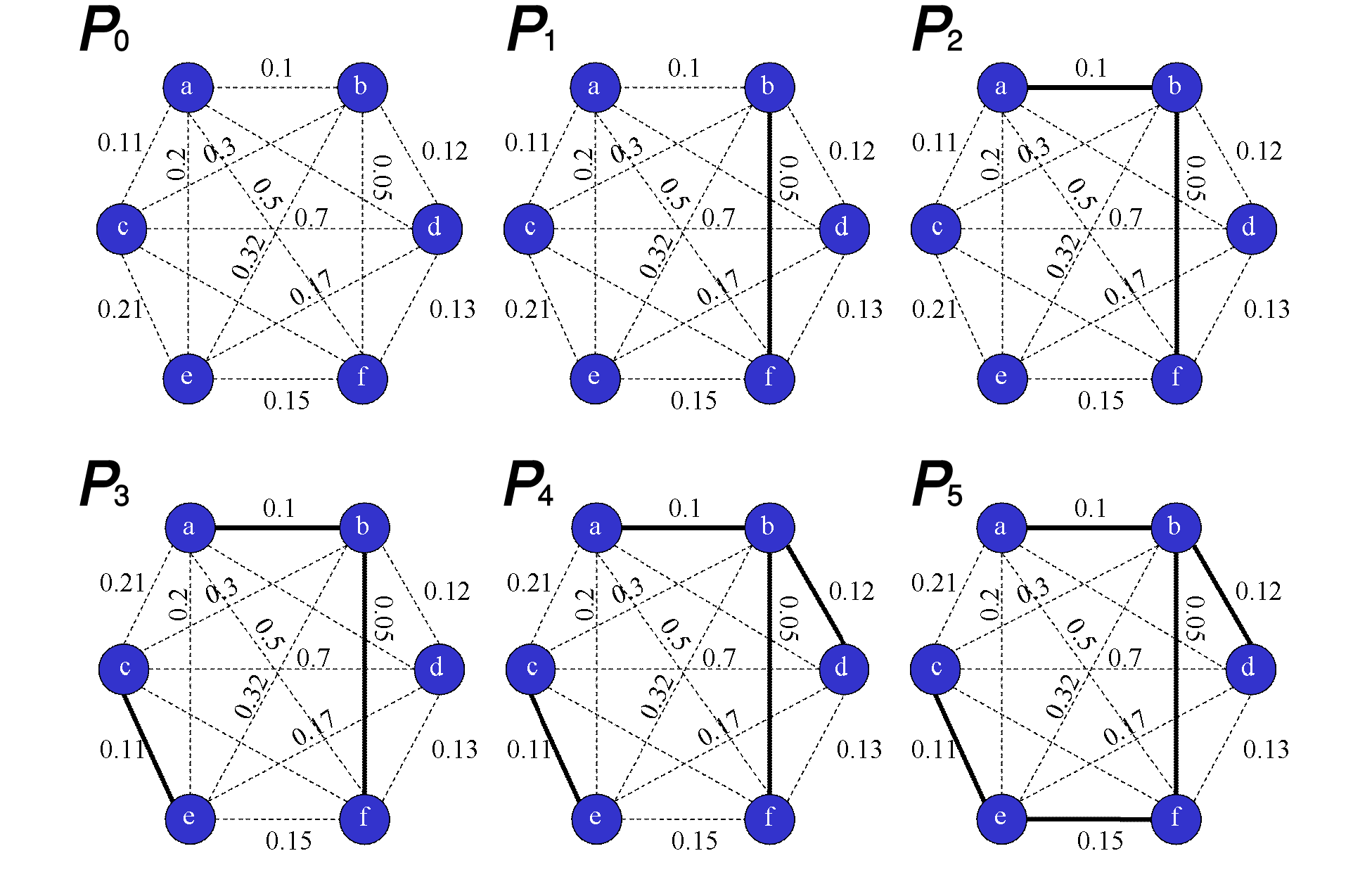

Supplement: Figure S3 — Illustration of the Kruskal algorithm. (TIF) [file pone.0017445.s003.tif]

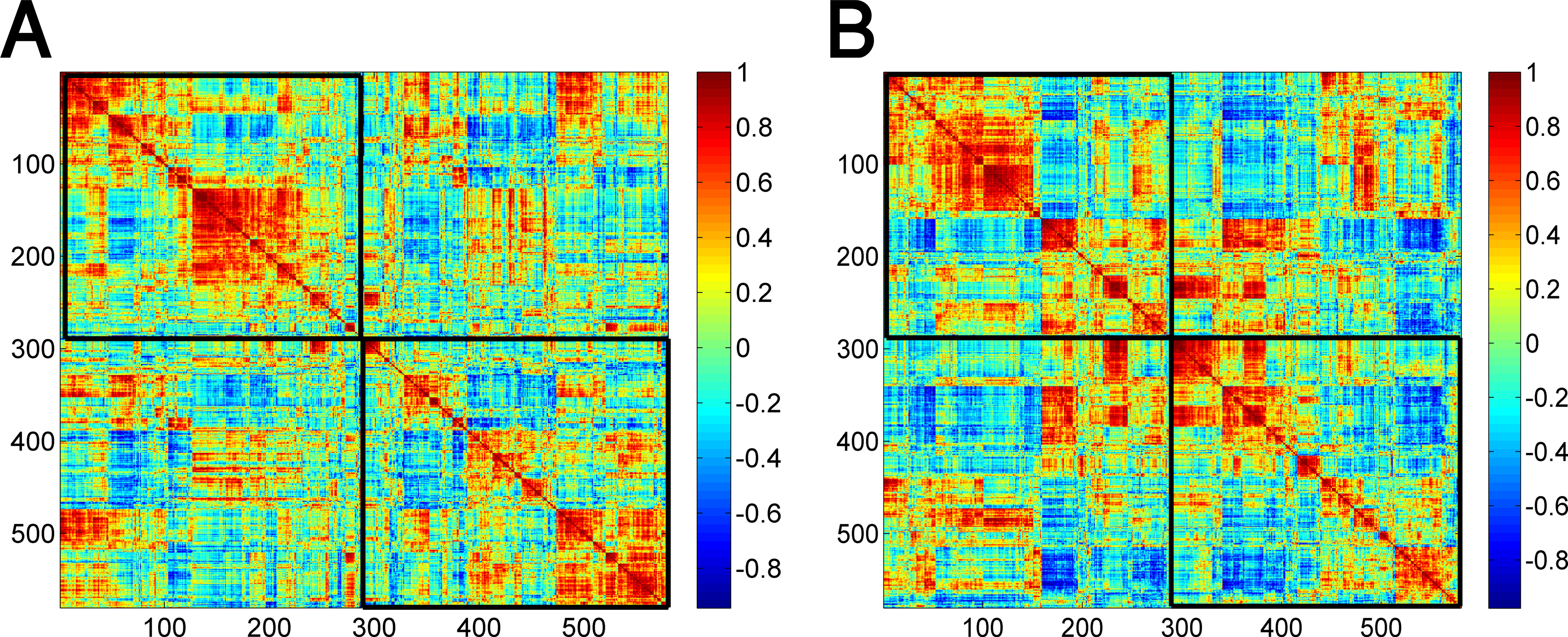

Supplement: Figure S4 — The integrated correlation matrices of the IgM and IgG datasets. Shown for (A) the mothers and (B) the cords. In each matrix, the IgM isotypes are in the top left frame and the IgG isotypes are in the bottom right frame. Both frames (isotypes) are ordered using a dendrogram algorithm demonstrating the relationships between correlated groups of antibodies of both isotypes. (TIF) [file pone.0017445.s004.tif]

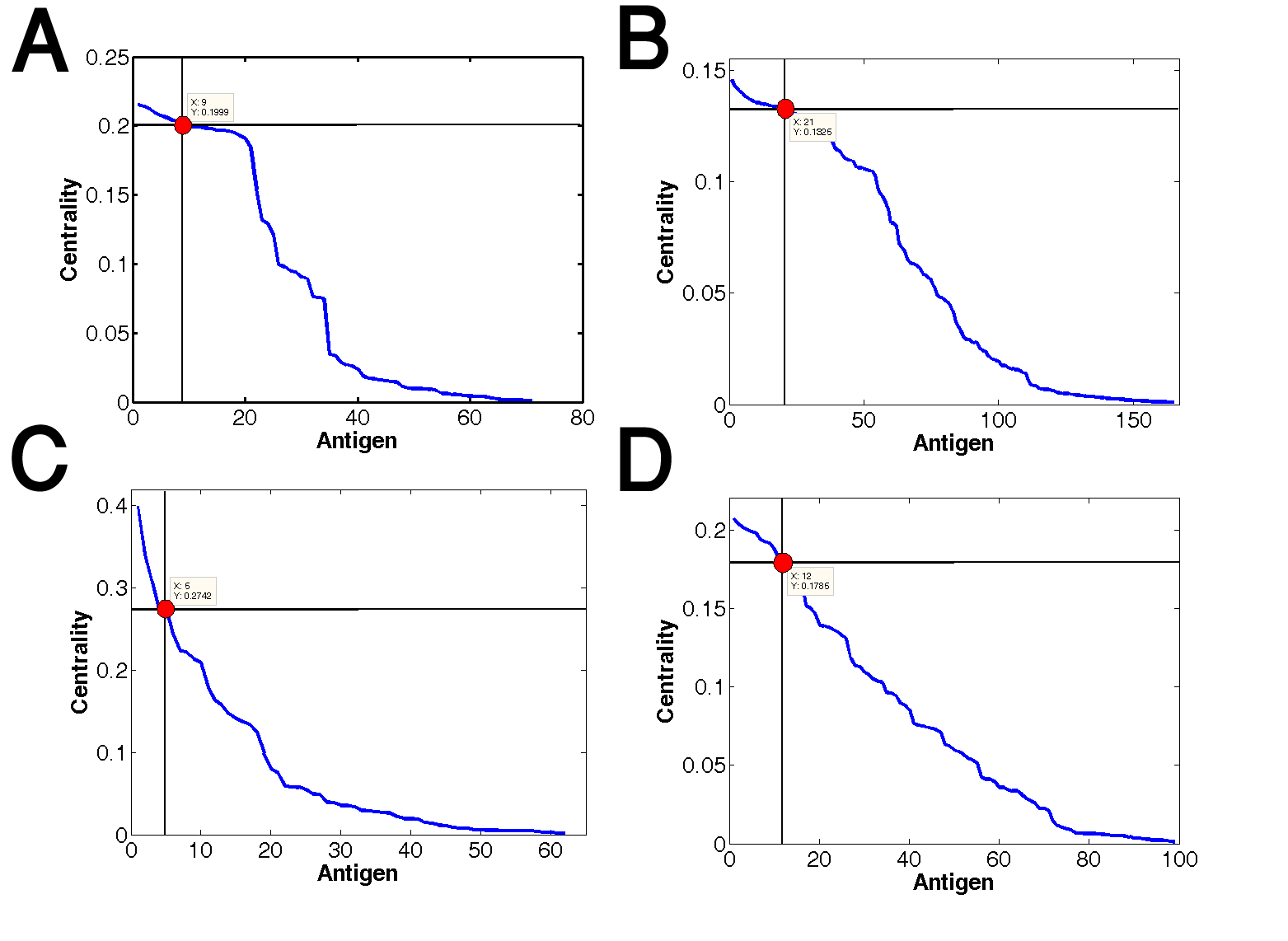

Supplement: Figure S5 — Antigen centrality values in descending order. Calculated for the maternal IgM (A), cord IgM (B), maternal IgG (C), and cord IgG (D). Marked in red dots are the numbers of antigens whose centrality values constitute 30 percent of the total centrality values in the network. Note that for clearer visualization zero values were removed prior to plotting the data. (TIF) [file pone.0017445.s005.tif]

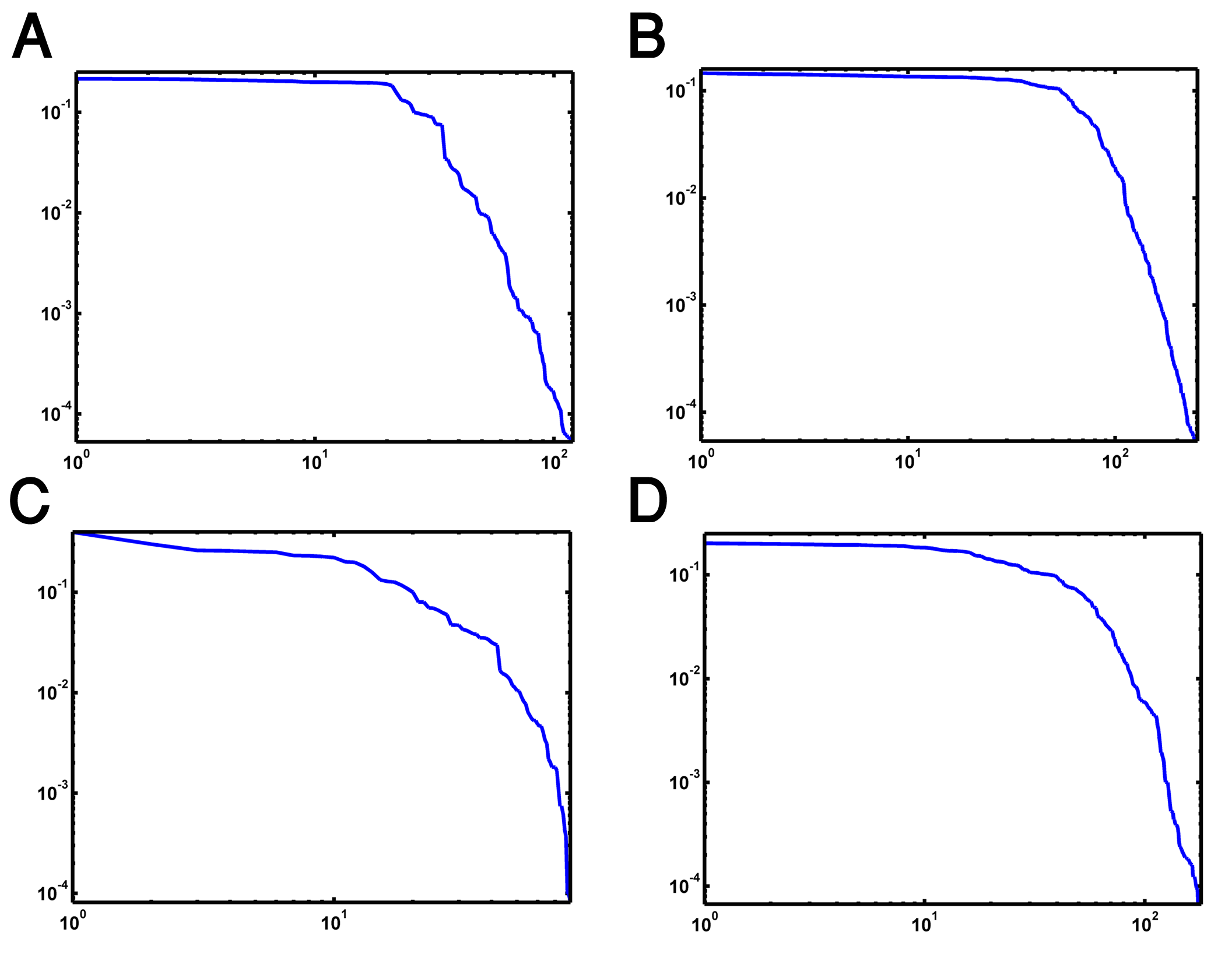

Supplement: Figure S6 — Zipf plots of node centrality values. Presented in descending order for the maternal IgM (A), cords' IgM (B), maternal IgG (C), and cords' IgG (D). Note that zero values were removed prior to plotting the data and the plots are presented in log scale. (TIF) [file pone.0017445.s006.tif]

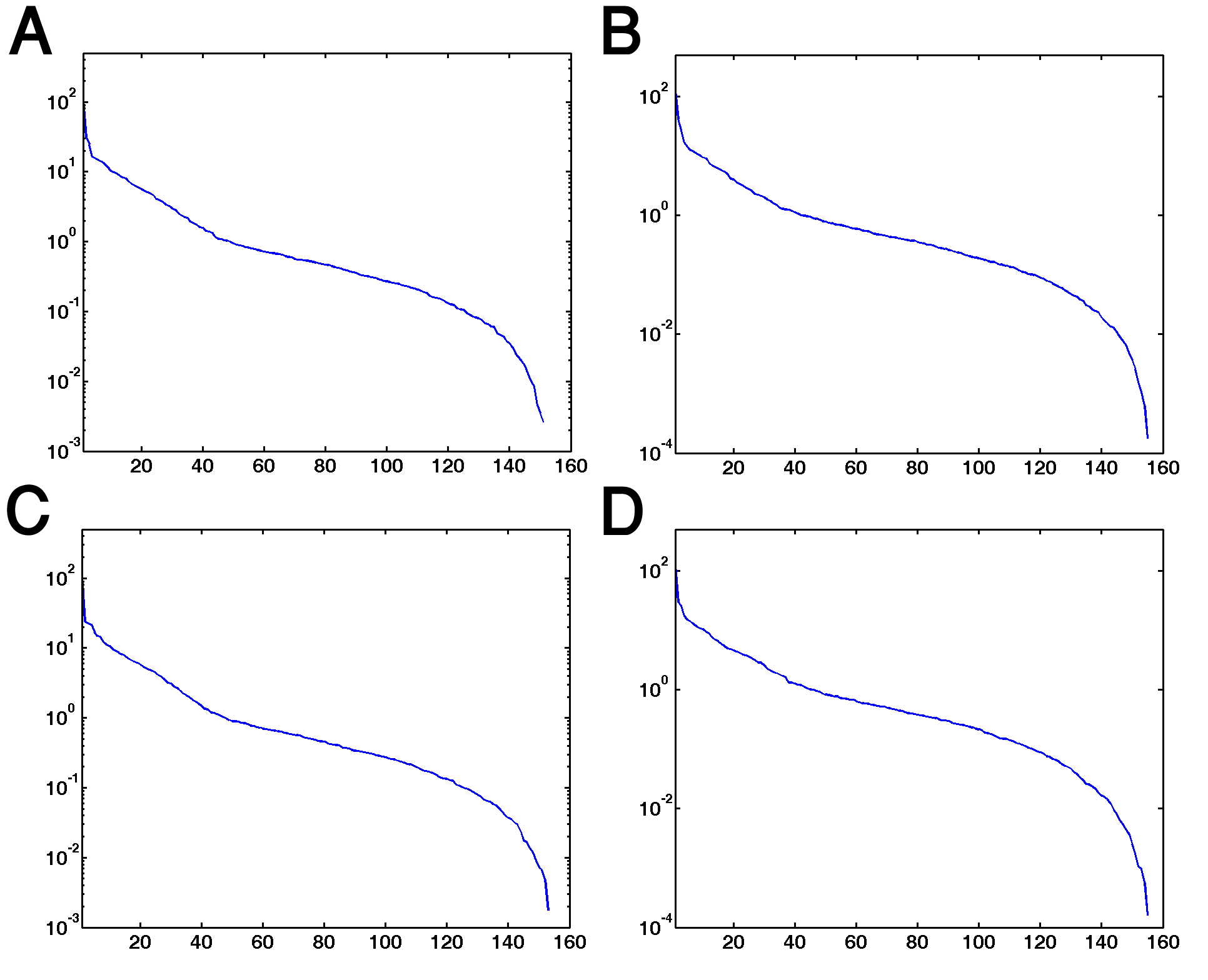

Supplement: Figure S7 — Semi-log plots of node centrality values. Presented in descending order for the maternal IgM (A), cords IgM (B), maternal IgG (C), and cords IgG (D). Note that zero values were removed prior to plotting the data and the plots are presented in semi-log scale. (TIF) [file pone.0017445.s007.tif]

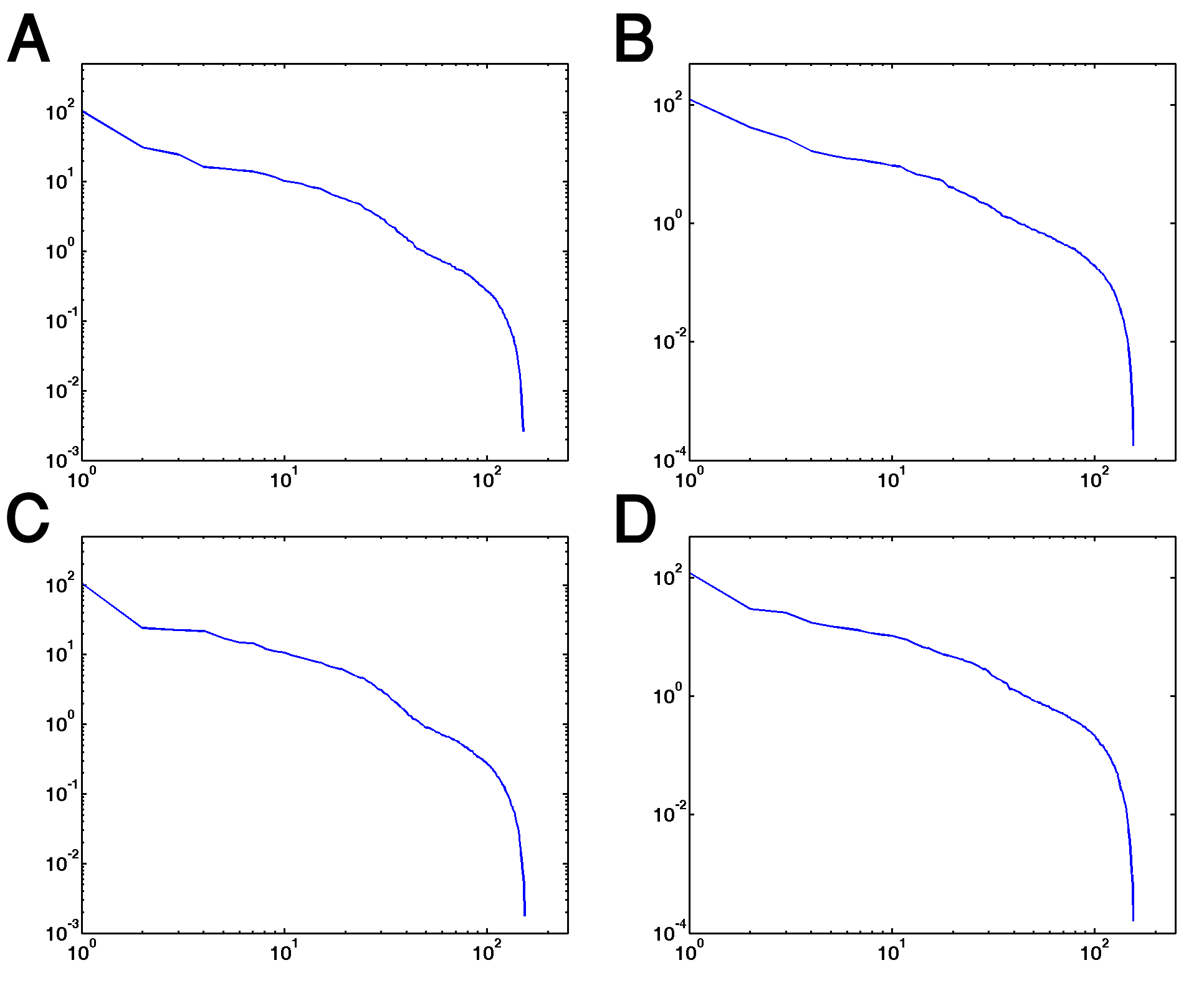

Supplement: Figure S8 — Zipf plots of the descending sorted eigenvalues of the correlation matrices (absolute values). Calculated for the maternal IgM (A), cords IgM (B), maternal IgG (C), and cords' IgG (D). Note that the plots are presented in log scale. (TIF) [file pone.0017445.s008.tif]

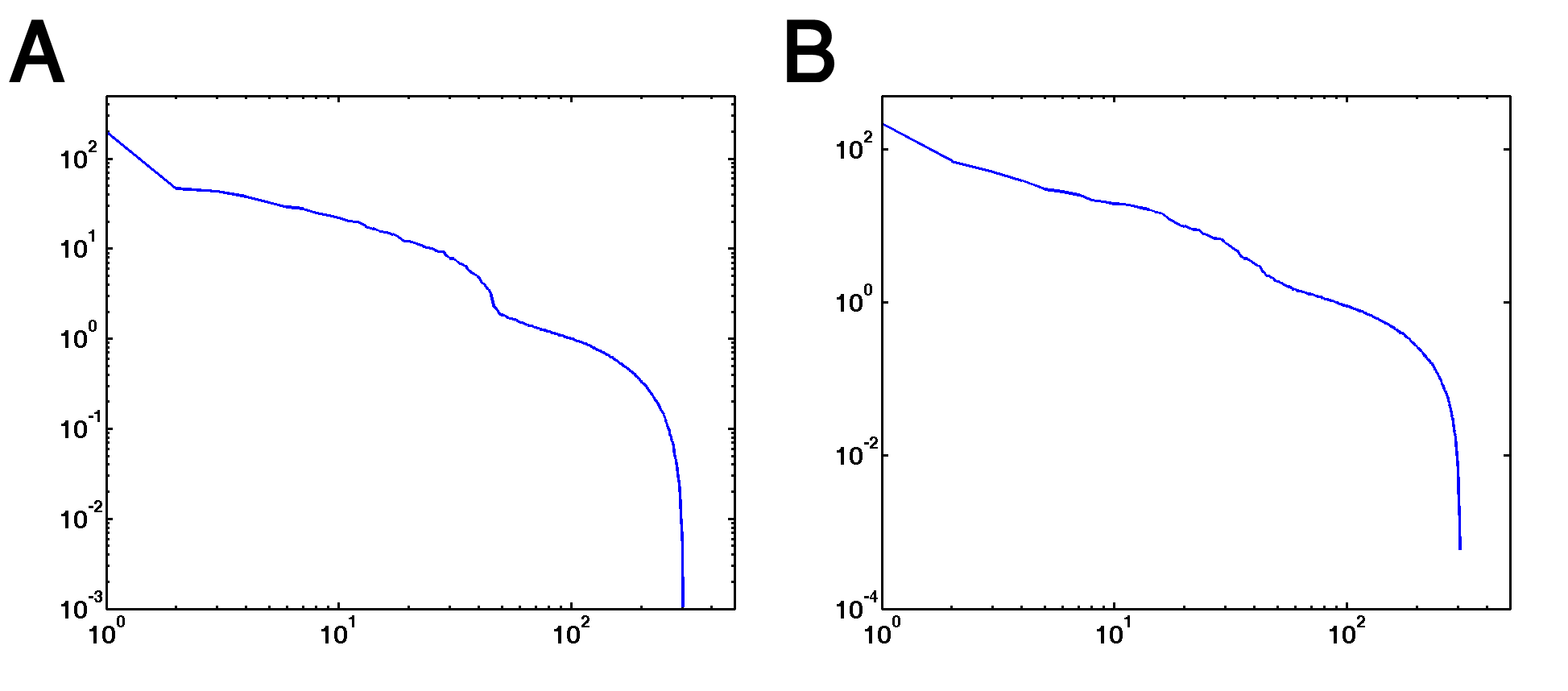

Supplement: Figure S9 — Zipf plots of the descending sorted eigenvalues of the correlation matrices (absolute values). Calculated for the maternal IgG and IgM (A), cords IgG and IgM (B). Note that the plots are presented in log scale. (TIF) [file pone.0017445.s009.tif]

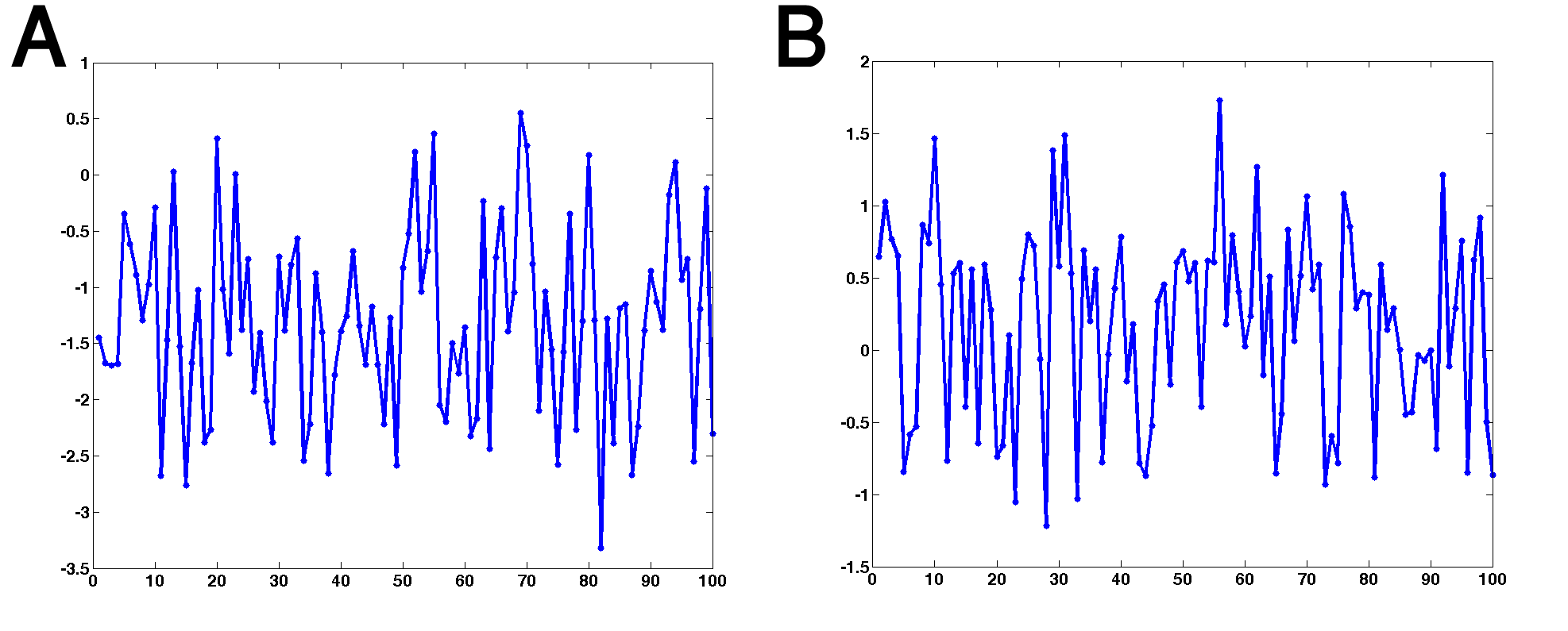

Supplement: Figure S10 — Robustness of the trees. We calculated the distance from all nodes to all others and subtracted it from the original calculated distance (before removal of the random nodes), this process was repeated 100 times and the results were plotted for (A) the maternal IgG and (B) maternal IgM. (TIF) [file pone.0017445.s010.tif]

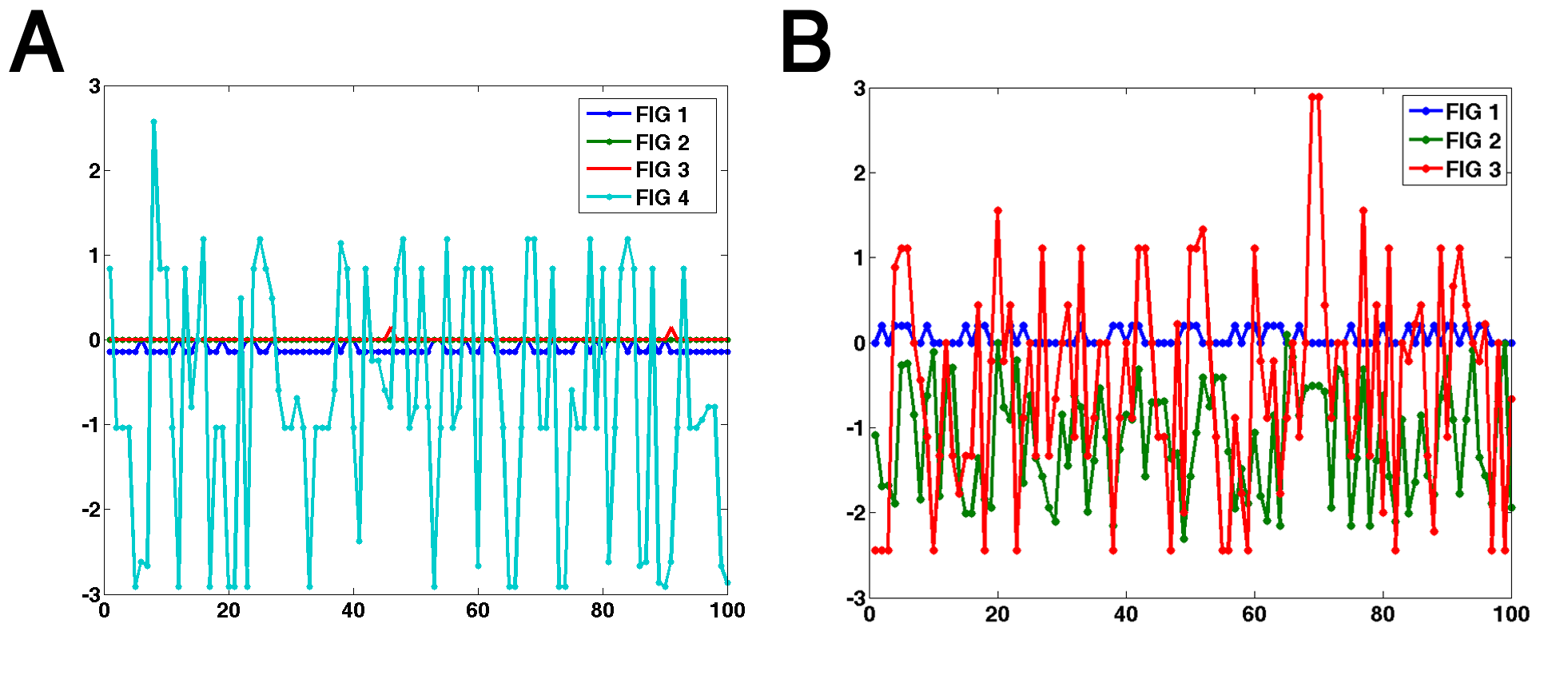

Supplement: Figure S11 — Maternal conservation of FIGs. For each of these randomly “trimmed” trees, we also calculated the average distance between all the nodes (members) within each FIG and subtracted it from the original calculated distance (before removal of the random nodes). (A) maternal IgM and (b) maternal IgG. (TIF) [file pone.0017445.s011.tif]
