# Supplementary material for: Network Theory Analysis of Antibody-Antigen Reactivity Data: The Immune Trees at Birth and Adulthood
Source: PLoS One. 2011 Mar 8;6(3):e17445. doi: 10.1371/journal.pone.0017445 (PMC3050881; doi:10.1371/journal.pone.0017445)
Supplement: Supporting Information S1 — Complete list of antigens spotted on the antigen chip. (PDF) [file pone.0017445.s012.pdf]

Supplemental Table 1. Complete list of antigens spotted on the antigen chip.

**Antigen**	**Description**	**Company**	**CAT #**
a2-macroglobulin		Sigma	M6159
Acetyl cholinesterase		Sigma	C2629
Acid Phosphatase	human prostatic	Sigma	P1649
a-Cristallyn		Sigma	C4163
Actin	bovine muscle	Sigma	A3653
Albumin	Bovine, fraction V	Sigma	A9647
Aldolase		Sigma	A8811
a-MSH	a-Melanocyte Stimulating Hormone	Sigma	M4135
Annexin 33 kDa	hum placenta	Sigma	A9460
Annexin 67 kDa	bovine liver	Sigma	A2824
ANP	Atrial Natriuretic Peptide, human	Sigma	A1663
Anti human IgA -Cy 5	Goat	Enco	109-175-011
Anti human IgG+ IgM -Cy 3		Enco	109-165-127
Anti human IgM	Goat	Sigma	I0759
Anti human IgM -Cy 5	Goat	Enco	109-175-043
Anti mouse IL-4	Purified, (capture)	Bactolab	PMG554434
b2-microglobulin		Sigma	M4890
b-Cristallin		Sigma	C6664
Beta Amyloid 1-20	Beta amyloid fragment 1-20	Sigma	A7723
Big Gastrin	I, human	Sigma	G5024
Biot. Anti-mouse IL-12 P40/P70		Bactolab	PMG554476
Beta Amyloid 17-40	Beta amyloid fragment 17-40	Sigma	A4848
b-MSH	b-Melanocyte Stimulating Hormone	Sigma	M6613
BNP	Brain Natriuretic Peptide	Sigma	B6900
BSA	Serum Albumin, bovine	Sigma	A7030

C protein	C-Reactive Protein	Sigma	C4063
C1Q	Complement C1Q, human	Sigma	C1740
C9	Complement C9, human	Sigma	C3660
CA19-9		Enco	770962
Cardiolipin	from bovine heart	Sigma	C0563
Cardiotoxin		Sigma	C3987
Cartilage Extract	Bovine articular crt.	Sigma	C5210
Caspase 3	human	Sigma	C1224
Caspase 8	human	Sigma	C1099
Catalase	bovine liver	Sigma	C9322
Cholesterol	water soluble	Sigma	C1145
Chorionic Gonadotrophin	human	Sigma	C8554
c-myc		Enco	26292-PO
Collagen II	bovine	Sigma	C1188
Collagen III	from human placenta	Sigma	C4407
Collagen IV	from human placenta	Sigma	C7521
Collagen IX	from human placenta	Sigma	C3657
Collagen VI	from human placenta	Sigma	C7521
Collagen VIII	from human placenta	Sigma	C7774
Collagen X	from human placenta	Sigma	C4407
Collagenase	type 1A	Sigma	C9891
C-peptide	human	Sigma	C5051
CRF	Corticotropin Releasing Factor, human, rat	Sigma	C3042
CTLA4		Enco	PHR5001
Cyclin	Cyclin A	Enco	SC-4072-WB
Cyclophosphamide, mono		Sigma	C0768
Cytochrome C P450	bovine heart	Sigma	C3131

Cytokeratin 8		Enco	771022
Cytokeratin 18		Enco	771032
DAP	Diabetes Associated Peptide amide	Sigma	D2162
Defensin		Enco	HDEFA15-R-10
Digitonin		Sigma	D141
dsDNA	double stranded DNA	Sigma	D1501
EGF	human, recom	Enco	PHG0064
elastase	from human neutrophils	Enco	191337
Endoproteinase	Endoproteinase Clu-c, S. aureus	Sigma	P6181
Endothelin 1	human, porcine	Sigma	E7764
Endothelin 2		Sigma	E9012
Enolase	rabbit muscle	Sigma	E0379
Factor II	Clotting Factor II, human	Sigma	F5132
Factor VII	Clotting Factor VII, (proconvertin) human	Enco	194191
Factor X	Clotting Factor X, human	Enco	194195
Fetuin		Sigma	F2379
Fibrin	washed from human plasma	Sigma	F5386
Fibrinogen	hum plasma, essentially plasminogen free	Sigma	F3879
Fibronectin	.1% solution	Sigma	F0895
GAD	Glutamic Acid Decarboxylase	Sigma	G2001
GAD/p34	>80% purirty	Sigma	
GAD/p35	>80% purirty	Sigma	
galectin 3	human	Cytolab	450-38-10
Galectin1(Gal-1)	human	Cytolab	450-39-10
Gastrin	human, I	Enco	152871
Gelsolin	from human plasma	Sigma	G1538
Gliadin	from wheat gluten	Enco	101778

Glucagon	human, bovine,porcine	Sigma	G1774
Glucocerebroside	from human spleen	Sigma	G9884
glyceraldehyde3phosphate dehydrogenase		Enco	100693
g-MSH	g-Melanocyte Stimulating Hormone	Sigma	M9638
GNRH	Growth Hormone Releasing Factor, human	Sigma	G8895
GpC	>80% purirty	Sigma	
GroEL (chaperonin 60E)	65 kDa heat shock protein, E. coli	Sigma	C7688
GroEL/p1	/ peptide 1	Sigma	
GroEL/p10	/ peptide 10	Sigma	
GroEL/p11	/ peptide 11	Sigma	
GroEL/p12	/ peptide 12	Sigma	
GroEL/p13	/ peptide 13	Sigma	
GroEL/p14	/ peptide 14	Sigma	
GroEL/p15	/ peptide 15	Sigma	
GroEL/p16	/ peptide 16	Sigma	
GroEL/p17	/ peptide 17	Sigma	
GroEL/p18	/ peptide 18	Sigma	
GroEL/p19	/ peptide 19	Sigma	
GroEL/p2	/ peptide 2	Sigma	
GroEL/p20	/ peptide 20	Sigma	
GroEL/p21	/ peptide 21	Sigma	
GroEL/p22	/ peptide 22	Sigma	
GroEL/p23	/ peptide 23	Sigma	
GroEL/p24	/ peptide 24	Sigma	
GroEL/p25	/ peptide 25	Sigma	
GroEL/p26	/ peptide 26	Sigma	
GroEL/p28	/ peptide 28	Sigma	

GroEL/p29	/ peptide 29	Sigma	
GroEL/p3	/ peptide 3	Sigma	
GroEL/p30	/ peptide 30	Sigma	
GroEL/p31	/ peptide 31	Sigma	
GroEL/p32	/ peptide 32	Sigma	
GroEL/p33	/ peptide 33	Sigma	
GroEL/p34	/ peptide 34	Sigma	
GroEL/p35	/ peptide 35	Sigma	
GroEL/p36	/ peptide 36	Sigma	
GroEL/p37	/ peptide 37	Sigma	
GroEL/p4	/ peptide 4	Sigma	
GroEL/p5	/ peptide 5	Sigma	
GroEL/p6	/ peptide 6	Sigma	
GroEL/p7	/ peptide 7	Sigma	
GroEL/p8	/ peptide 8	Sigma	
GroEL/p9	/ peptide 9	Sigma	
GST	Glutathione-S-Transferase, hum	Sigma	G8642
GSTase	Galactosyltransferase	Sigma	G5507
HDL	Lipoprotein, High Density	Sigma	L1567
Hemaglutinin	fillamentous	Sigma	F5551
Hemoglobin	ferrous, hum	Sigma	H0267
Heparin	porcine intestinal mucosa	Sigma	H3393
hGST	Glutathion-S-transferase human	Sigma	G8842
Histone IIA	unfractionated whole	Sigma	H9250
holo-transferase		Enco	152335
HSP27	human recombinant	Enco	ESP-715G
HSP40	recomb	Enco	SPP-400B

HSP 47	rat, recomb.	Sigma	H8035
HSP60/batch 2	/ batch 2	Sigma	H8903
HSP60/p1	/ peptide 1	Sigma	
HSP60/p10	/ peptide 10	Sigma	
HSP60/p12	/ peptide 12	Sigma	
HSP60/p14	/ peptide 14	Sigma	
HSP60/p16	/ peptide 16	Sigma	
HSP60/p18	/ peptide 18	Sigma	
HSP60/p19	/ peptide 19	Sigma	
HSP60/p2	/ peptide 2	Sigma	
HSP60/p22	/ peptide 22	Sigma	
HSP60/p23	/ peptide 23	Sigma	
HSP60/p25	/ peptide 25	Sigma	
HSP60/p26	/ peptide 26	Sigma	
HSP60/p27	/ peptide 27	Sigma	
HSP60/p277	/ peptide p277	Sigma	
HSP60/p28	/ peptide 28	Sigma	
HSP60/p29	/ peptide 29	Sigma	
HSP60/p30	/ peptide 30	Sigma	
HSP60/p32	/ peptide 32	Sigma	
HSP60/p33	/ peptide 33	Sigma	
HSP60/p34	/ peptide 34	Sigma	
HSP60/p35	/ peptide 35	Sigma	
HSP60/p36	/ peptide 36	Sigma	
HSP60/p37	/ peptide 37	Sigma	
HSP60/p38	/ peptide 38	Sigma	
HSP60/p4	/ peptide 4	Sigma	

HSP60/p5	/ peptide 5	Sigma	
HSP60/p6	/ peptide 6	Sigma	
HSP60/p7	/ peptide 7	Sigma	
HSP60/p8	/ peptide 8	Sigma	
HSP60/p9	/ peptide 9	Sigma	
HSP60p/24	/ peptide 24	Sigma	
HSP65	65 kDa heat shock protein, (M. tuberculosis) M. bovis instead	Enco	NSP-581E
HSP65/p180	/ peptide 180	Sigma	
HSP65/p278	/ peptide 278	Sigma	
HSP65/p3	/ peptide 3	Sigma	
HSP70	70 kDa heat shock protein, human	Sigma	H8778
HSP70/p10	/ peptide 10	Sigma	
HSP70/p11	/ peptide 11	Sigma	
HSP70/p12	/ peptide 12	Sigma	
HSP70/p13	/ peptide 13	Sigma	
HSP70/p14	/ peptide 14	Sigma	
HSP70/p17	/ peptide 17	Sigma	
HSP70/p18	/ peptide 18	Sigma	
HSP70/p2	/ peptide 2	Sigma	
HSP70/p20	/ peptide 20	Sigma	
HSP70/p22	/ peptide 22	Sigma	
HSP70/p23	/ peptide 23	Sigma	
HSP70/p24	/ peptide 24	Sigma	
HSP70/p26	/ peptide 26	Sigma	
HSP70/p28	/ peptide 28	Sigma	
HSP70/p29	/ peptide 29	Sigma	

HSP70/p3	/ peptide 3	Sigma	
HSP70/p30	/ peptide 30	Sigma	
HSP70/p31	/ peptide 31	Sigma	
HSP70/p32	/ peptide 32	Sigma	
HSP70/p33	/ peptide 33	Sigma	
HSP70/p36	/ peptide 36	Sigma	
HSP70/p37	/ peptide 37	Sigma	
HSP70/p4	/ peptide 4	Sigma	
HSP70/p5	/ peptide 5	Sigma	
HSP70/p6	/ peptide 6	Sigma	
HSP70/p8	/ peptide 8	Sigma	
HSP70/p9	/ peptide 9	Sigma	
HSP71	71 kDa heat shock protein, M. tuberculosis	Enco	SPP-885B
HSP90	from bovine brain	Sigma	H6774
human Albumin	Serum Albumin, human, globulin free	Sigma	A8763
human IgG	tech grade	Sigma	I8664
human IgM	reagent grade	Sigma	I8260
IFNg	Interferong, hum recomb	Sigma	I3265
IGFBP	Insulin-like Growth Factor Binding Protein 2	Sigma	I5403
IL-10	Interleukin-10	Cytolab	200-10-2
IL-12	Interleukin-12	Cytolab	200-12-2
IL-15	Interleukin-15	Cytolab	200-15-2
IL-2	Interleukin-2, human	Cytolab	200-02-10
IL21	recomb.	Enco	PHC0214
IL-2R a-chain/p1	IL-2 Receptor a-chain/peptide 1	Sigma	
IL-2R a-chain/p2	IL-2 Receptor a-chain/peptide 2	Sigma	
IL-2R b-chain/p1	IL-2 Receptor b-chain / peptide 1	Sigma	

IL-4	Interleukin-4, human	Cytolab	200-04-2
IL-5	Interleukin-5, human	Cytolab	200-05-2
IL-6	Interleukin-6, human	Cytolab	200-06-2
IL-8	h rec	Sigma	I 1645
Insulin	HUMAN RECOMBINANT	Sigma	I0259 I2643
Insulin chain A	oxidized ammonium salt	Sigma	I1633
Insulin chain B	oxidized from bovine pancreas	Sigma	I6383
KLH	Keyhole Lympet Hemocyanin	Sigma	H7017
Lactoferrin		Enco	151535
Laminin	from human placenta	Sigma	L6274
LDL	Lipoprotein, Low Density	Sigma	L7914
LHRH	Luteinizing Hormone-Releasing Hormone	Sigma	L4897
Lipid A sm	Lipid A, S. minessotta	Sigma	L6895
LPS	E. coli	Sigma	L3755
MAGE1		Enco	PRO-311 (A)
MART1		Enco	PRO-299
MBP	Myelin Basic Protein, guinea pig	Sigma	M2295
MBP rat	Myelin Basic Protein, rat	Enco	H-1964.0001
melanin	Melanin concentrating hormone	Sigma	M4542
Melanostatin	Pro-Leu_Gly amide	Sigma	P9887
melatonin	Crystalline	Sigma	M5250
met BSA	methylated Serum Albumin, bovine	Sigma	A1009
MIF		Enco	4282-PO1
Mig (CXCL9)	monokine induced by interferon gamma	Enco	PHC1604
MMP1	human tissue	Sigma	M1802
MMP2		Sigma	M7942
MMP3		Sigma	M1677

MMP9		Sigma	M4809
MOBP/p78-89	Myelin-Associated Oligodendrocytic Basic Protein	Sigma	
MOG	Myelin Oligodendrocyte Glycoprotein, mouse	Enco	H-4184-0500
MOG/p35-55	Myelin Oligodendrocyte Glycoprotein, mouse/p35-55	Enco	H5912-0500
MOG/p94-116	Myelin Oligodendrocyte Glycoprotein, mouse/p94-116	Sigma	
mouse IgG	technical grade	Sigma	I8765
Mouse IgM, Kappa	ABPC22	Sigma	M3795
Mouse IL-10	recomb.	Bactolab	PMG550070
Mouse IL-12	Recombinant	Bactolab	PG554592
Mouse IL-12, P40	Recombinant	Bactolab	PMG554594
MUC1	mucin	Enco	155742
Myeloperoxidase	hum leucocytes	Sigma	M6908
Myoglobin	human heart	Sigma	M6036
Myosin	(bovine muscle) rabbit muscle instead	Sigma	M6643?? M1836
Neuropeptide Y		Sigma	N5017
Neurotensin acetate		Sigma	N6383
OVA	Ovoalbumin	Sigma	A5378
Oxytocin		Sigma	O3251
Pepstatin	A, nicrobial source	Sigma	P5318
Peroxidase	horseradish	Sigma	P6782
Phosphatydil-ethanol-amine	Type 1	Sigma	P9137
Phospholipase D		Sigma	P4912
Plasmin		Sigma	P1867
PLP	Proteolipid Protein	Enco	6420-5150
Poly arginine		Sigma	P3892

Poly D- lysine		Sigma	P4408
Poly L-aspartic acid		Sigma	P6762
Poly L-glutamate		Sigma	P4636
PPD	Purified Protein Derivative, M. tuberculosis	Enco	P-1000-001
Proinsulin		Enco	VCBVB-612
Protamine Sulfate		Sigma	P4020
Protease 133		Enco	150210
PT	Pertussis Toxin	Sigma	P2980
PTH	Parathyroid Hormone	Sigma	P7036
PTHrP		Enco	H-6630-0.500
Rat anti mouse IL-10	Purified, biotinilated	Bactolab	PMG554423
rat IgG		Sigma	I4131
Ribonuclease		Sigma	R4875
SOD	Super Oxide Dismutase	Sigma	S9636
Somatostatin		Sigma	S1763
Spectrin	hum erythrocytes	Sigma	S3644
ssDNA	single stranded DNA	Sigma	D8899
Substance P		Sigma	S6883
Synuclein	alpha	Enco	H00006622-PO1
TCR bchain/pC2C	/ peptide C2C	Sigma	
TCR bchain/pMED12	/ peptide MED12	Sigma	
TCR bchain/pN12	TCR bchain / peptide N12	Sigma	
TCR-CDR3-pC9		Sigma	
TGF-beta		Enco	PHG9104
Thrombin	human plasma	Sigma	T6884
Thyrocalcitonin	human	Sigma	T3535
Thyroglobulin	bovine	Sigma	T1001

TNF	Tumor Necrosis Factor - alpha	Enco	PHP051
TNFR	Tumor Necrosis Factor Receptor,h recomb.	Enco	PHP131
Transferrin	human, holo-transferrin	Sigma	T4132
Transglutaminase	human reco	Enco	PRO-308 (A)
Tropomyosin	bovine muscle	Sigma	T2400
Troponin	from porcine muscle	Sigma	T2275
Tubulin		Enco	9280-3050
Tyrosinase	mushroom	Sigma	T3824
Ubiquitin	bovine RBC	Sigma	U6253
Vasopresin		Sigma	V0377
Vimentin	bovine lens	Sigma	V4383
VIP	Vasointestinal Peptide	Enco	064-21
Vitronectin	from human plasma	Sigma	V8379
