# Supplementary material for: Network Theory Analysis of Antibody-Antigen Reactivity Data: The Immune Trees at Birth and Adulthood
Source: PLoS One. 2011 Mar 8;6(3):e17445. doi: 10.1371/journal.pone.0017445 (PMC3050881; doi:10.1371/journal.pone.0017445)
Supplement: Supporting Information S2 — The eigen-values' entropy of the correlation matrices. (DOC) [file pone.0017445.s013.doc]

**Supporting Information**

**Network Theory Analysis of Antibody-Antigen Reactivity Data: The Immune Trees at Birth and Adulthood**

Asaf Madi^1,2,*^, Dror Y. Kenett^1,*^, Sharron Bransburg-Zabary^1,2^, Yifat Merbl^3,4^, Francisco J. Quintana^3,5^, Alfred I. Tauber^6^, Irun R. Cohen^3,#^, and Eshel Ben-Jacob^1,7,#^

**Supporting Information S2: The eigen-values' entropy of the correlation matrices**

To compare the information embedded in the MST and the eigenvalue statistics of the correlation matrices, we calculated the eigenvalue entropies [47], defined by,

$S\equiv-\frac{1}{\log\left(N\right)}\sum_{i=1}^{N} \Omega(i)\log\left[\Omega(i)\right]$ (S-1)

where $\Omega$ is given by,

$\Omega\left(i\right)=\frac{\lambda\left(i\right)^{2}}{\sum_{i=1}^{N} \lambda\left(i\right)^{2}}$ (S-2)

and λ(i) denotes the matrix eigenvalue. Note that the $1/\log(N)$normalization was selected to ensure that S = 1 for the maximum eigenvalue entropy limit of a uniform eigenvalue distribution. We note that, since maximal entropy equals one, the latent information LI is associated with 1-S, so it is higher for lower entropies.

**Table S2:** Entropy calculations for the absolute values of the correlation matrices

|  | maternal | cords |
| --- | --- | --- |
| IgM | 0.236 | 0.186 |
| IgG | 0.204 | 0.172 |
| IgM and IgG | 0.221 | 0.203 |

**Table S3:** Entropy calculations for the absolute values of the affinity matrices

|  | maternal | cords |
| --- | --- | --- |
| IgM | 0.191 | 0.159 |
| IgG | 0.150 | 0.154 |
| IgM and IgG | 0.172 | 0.159 |

**Table S4:** Entropy calculations for the shifted values of the correlation matrices

|  | maternal | cords |
| --- | --- | --- |
| IgM | 0.083 | 0.069 |
| IgG | 0.066 | 0.081 |
| IgM and IgG | 0.069 | 0.075 |

**Table S5:** Entropy calculations for the values of the affinity matrices

|  | maternal | cords |
| --- | --- | --- |
| IgM | 0.082 | 0.070 |
| IgG | 0.060 | 0.079 |
| IgM and IgG | 0.066 | 0.077 |

**Table S6:** Entropy calculations for the centrality values (after removal of zeros)

|  | maternal | cords |
| --- | --- | --- |
| IgM | 0.633 | 0.731 |
| IgG | 0.740 | 0.697 |

Entropy calculations for the shifted values of the matrices, please note that while the correlation values were shift from [-1,1] to [0,1] the affinity values were not (due to the fact that they are already in this spectrum.
