# Supplementary material for: Network Theory Analysis of Antibody-Antigen Reactivity Data: The Immune Trees at Birth and Adulthood
Source: PLoS One. 2011 Mar 8;6(3):e17445. doi: 10.1371/journal.pone.0017445 (PMC3050881; doi:10.1371/journal.pone.0017445)
Supplement: Supporting Information S3 — Calculation of the node centrality. (DOC) [file pone.0017445.s014.doc]

**Supporting Information**

**Network Theory Analysis of Antibody-Antigen Reactivity Data: The Immune Trees at Birth and Adulthood**

Asaf Madi^1,2,*^, Dror Y. Kenett^1,*^, Sharron Bransburg-Zabary^1,2^, Yifat Merbl^3,4^, Francisco J. Quintana^3,5^, Alfred I. Tauber^6^, Irun R. Cohen^3,#^, and Eshel Ben-Jacob^1,7,#^

**Supporting Information S3: Calculation of the node centrality**

To calculate the eigenvalue centrality, one must first filter the correlation matrix using a chosen threshold. Determining which threshold to use is not a simple task. Here we have chosen to use a measure of normalized STD of the principal eigenvector.

We begin by choosing a correlation threshold, and use it to create an adjacency matrix, where correlations above the threshold correspond to 1 in the adjacency matrix, and correlations below the threshold correspond to 0. We diagonalize the adjacency matrix, and focus on the principal eigenvector (the eigenvector corresponding to the largest eigenvalue). The centrality of each variable is defined as the weight of each variable in the principal eigenvector. We then compute the STD of the components for the principal eigenvector, and normalize it by the number of variables non-zero component. Doing so allows us to search for a threshold that gives a compromise between high values of centrality, and a small number of variables, and in so doing we can identify a significant sub-group of variables with the highest centrality in the network.

We calculate the normalized STD of the eigenvalue centrality for different thresholds, ranging from 0 to 0.95 correlations. In Figure S1 we present the normalized STD as a function of the correlation threshold, for the maternal IgM (A), cords’ IgM (B), maternal IgG (C), and cords’ IgG (D). We continued to study the resulting normalized STDs by investigating their second derivative (Figure S2), and have found that the fluctuations in the second derivative of the STD begin at thresholds above 0.79 and more specifically 0.82 for the maternal IgM, 0.82 for the cords’ IgM, 0.79 for the maternal IgG and 0.86 for the cords’ IgG.
