# Supplementary material for: Network Theory Analysis of Antibody-Antigen Reactivity Data: The Immune Trees at Birth and Adulthood
Source: PLoS One. 2011 Mar 8;6(3):e17445. doi: 10.1371/journal.pone.0017445 (PMC3050881; doi:10.1371/journal.pone.0017445)
Supplement: Supporting Information S4 — Construction of the immune trees by the Kruskal algorithm. (DOC) [file pone.0017445.s015.doc]

**Supporting Information**

**Network Theory Analysis of Antibody-Antigen Reactivity Data: The Immune Trees at Birth and Adulthood**

Asaf Madi^1,2,*^, Dror Y. Kenett^1,*^, Sharron Bransburg-Zabary^1,2^, Yifat Merbl^3,4^, Francisco J. Quintana^3,5^, Alfred I. Tauber^6^, Irun R. Cohen^3,#^, and Eshel Ben-Jacob^1,7,#^

**Supporting Information S4: Construction of the immune trees by the Kruskal algorithm**

Kruskal's algorithm is conceptually straight forward. The edges are selected and added to the spanning tree in increasing order of their weights. An edge is added to the tree only if it does not create a cycle. It builds the MST as a collection of small trees (forest). Initially, each vertex is considered as its own tree in the forest. Then, the algorithm considers each edge in turn, in order by increasing weight. If an edge (u, v) connects two different trees, then (u, v) is added to the set of edges of the MST, and two trees connected by an edge (u, v) are merged into a single tree on the other hand, if an edge (u, v) connects two vertices in the same tree, then edge (u, v) is discarded. This is repeated until the forest is reduced into one tree (Figure S3).

*P_0_* = {{a},{b},{c},{d},{e},{f}}

*P_1_* = {{a},{b, f},{c},{d},{e}}

*P_2_* = {{a, b, f},{c},{d},{e}}

*P_3_* = {{a, b, f},{c, e},{d}}

*P_4_* = {{a, b, f, d},{c, e}}
