# Supplementary material for: Network Theory Analysis of Antibody-Antigen Reactivity Data: The Immune Trees at Birth and Adulthood
Source: PLoS One. 2011 Mar 8;6(3):e17445. doi: 10.1371/journal.pone.0017445 (PMC3050881; doi:10.1371/journal.pone.0017445)
Supplement: Supporting Information S5 — The integrated correlation matrices. (DOC) [file pone.0017445.s016.doc]

**Supporting Information**

**Network Theory Analysis of Antibody-Antigen Reactivity Data: The Immune Trees at Birth and Adulthood**

Asaf Madi^1,2,*^, Dror Y. Kenett^1,*^, Sharron Bransburg-Zabary^1,2^, Yifat Merbl^3,4^, Francisco J. Quintana^3,5^, Alfred I. Tauber^6^, Irun R. Cohen^3,#^, and Eshel Ben-Jacob^1,7,#^

**Supporting Information S5: The integrated correlation matrices**

To test whether the IgM network might influence the IgG network, we analyzed the integrated correlation matrices of the mothers and cords (Figure S4) for the IgM and IgG datasets. For a better display of the associations between the isotypes, we ordered them using a dendrogram algorithm. This ordering helped demonstrate the relationships between correlated groups of antibodies of both isotypes.
