# Supplementary material for: Network Theory Analysis of Antibody-Antigen Reactivity Data: The Immune Trees at Birth and Adulthood
Source: PLoS One. 2011 Mar 8;6(3):e17445. doi: 10.1371/journal.pone.0017445 (PMC3050881; doi:10.1371/journal.pone.0017445)
Supplement: Supporting Information S6 — The topological statistics of the immune trees. (DOC) [file pone.0017445.s017.doc]

**Supporting Information**

**Network Theory Analysis of Antibody-Antigen Reactivity Data: The Immune Trees at Birth and Adulthood**

Asaf Madi^1,2,*^, Dror Y. Kenett^1,*^, Sharron Bransburg-Zabary^1,2^, Yifat Merbl^3,4^, Francisco J. Quintana^3,5^, Alfred I. Tauber^6^, Irun R. Cohen^3,#^, and Eshel Ben-Jacob^1,7,#^

**Supporting Information S6: The topological statistics of the immune trees**

To quantify the differences between the cord and maternal merged MSTs (Figure 3), we measured the topological distances between the different nodes in each tree, where distance is measured in terms of the number of edges. The results are presented in Table S7. Inspection of the node isotypic origin – the average distance between IgG/IgM nodes – reveals that there is a higher separation between the isotypes in the merged tree of the cords in comparison to that of the mothers. It appears that the maternal merged MST manifests greater isotypic integration in the mothers’ immune network in comparison to that of the newborns. This suggests that natural maturation of the immune system from the newborn state to young adulthood might involve strengthening of the immune tree architecture of the autoantibody repertoire by the evolution of more correlated antibody reactivities.

Furthermore, the average distance between two isotypes binding to the *same antigen* reveals that in general, such isotype pairs are not linked in the tree. Thus, the correlation between the patterns of two specific antigen reactivities is not necessarily due to the antigen that trigger that binds to them. Finally, the distances between all nodes indicate that, in general, the merged tree in the cord repertoire is wider and more dispersed than that of the mothers (Table S7).

Significance levels where determined using the following calculation:

$significance = \frac{\left| \overline{x} - \overline{y} \right|}{\frac{1}{2}\left(\sigma_{x}+\sigma_{y}\right)}$ (S-3)

**Table S7:** Comparison of topological measures for the cord and maternal IMST

|  | **Cords** | | **Mothers** | |  |
| --- | --- | --- | --- | --- | --- |
|  | **Average** | **STD** | **Average** | **STD** | **Significance** |
| Average distance between all nodes | 24.50 | 2.52 | 20.853 | 1.553 | 1.7908 |
| Maximum distance between nodes | 61 |  | 53 |  |  |
| Average distance between IgG nodes | 19.68 | 1.557 | 18.45 | 1.127 | 0.9165 |
| Average distance between IgM nodes | 22.838 | 2.25 | 22.193 | 1.968 | 0.3058 |
| Average distance between IgM and IgG nodes | 27.47 | 1.914 | 21.38 | 1.639 | 3.4271 |
| Average distance between every two isotypes of the same antigen | 28.196 | 13.73 | 20.81 | 10.72 | 0.6042 |
