# Supplementary material for: Network Theory Analysis of Antibody-Antigen Reactivity Data: The Immune Trees at Birth and Adulthood
Source: PLoS One. 2011 Mar 8;6(3):e17445. doi: 10.1371/journal.pone.0017445 (PMC3050881; doi:10.1371/journal.pone.0017445)
Supplement: Supporting Information S7 — The central nodes – the network hubs. (DOC) [file pone.0017445.s018.doc]

**Supporting Information**

**Network Theory Analysis of Antibody-Antigen Reactivity Data: The Immune Trees at Birth and Adulthood**

Asaf Madi^1,2,*^, Dror Y. Kenett^1,*^, Sharron Bransburg-Zabary^1,2^, Yifat Merbl^3,4^, Francisco J. Quintana^3,5^, Alfred I. Tauber^6^, Irun R. Cohen^3,#^, and Eshel Ben-Jacob^1,7,#^

**Supporting Information S7: The central nodes – the network hubs**

Figure S5 shows node centrality – hubs – in descending order for the IgG and IgM antigen-reactivity networks of the mothers and cords.

Zipf plots of the node centrality values (Figure S6):

Semi-log plots of the above datasets (Figure S7):

Figure S8 shows the Zipf plots of the descending sorted eigenvalues of the correlation matrices (absolute values):

Figure S9 shows the Zipf plots of the descending sorted eigenvalues of the correlation matrices of the integrated isotypes (absolute values):
