# Supplementary material for: Network Theory Analysis of Antibody-Antigen Reactivity Data: The Immune Trees at Birth and Adulthood
Source: PLoS One. 2011 Mar 8;6(3):e17445. doi: 10.1371/journal.pone.0017445 (PMC3050881; doi:10.1371/journal.pone.0017445)
Supplement: Supporting Information S8 — The immune tree robustness. (DOC) [file pone.0017445.s019.doc]

**Supporting Information**

**Network Theory Analysis of Antibody-Antigen Reactivity Data: The Immune Trees at Birth and Adulthood**

Asaf Madi^1,2,*^, Dror Y. Kenett^1,*^, Sharron Bransburg-Zabary^1,2^, Yifat Merbl^3,4^, Francisco J. Quintana^3,5^, Alfred I. Tauber^6^, Irun R. Cohen^3,#^, and Eshel Ben-Jacob^1,7,#^

**Supporting Information S8: The immune tree robustness**

Robustness of the trees: to test the robustness of the trees we removed 50 random antigens from the dataset and re-created the trees. We than calculated the distance from all nodes to all others and subtracted from the original calculated distance (before removal of the random nodes), this process was repeated 100 times and plotted the results in Figure S10.

In addition, for each of these randomly “trimmed” trees, we also calculated the average distance between all the nodes (members) within each FIG and subtracted it from the original calculated distance (before removal of the random nodes). The results show very high conservation for the maternal IgM dataset (Figure S11A) and good conservation for the maternal IgG dataset (Figure S11B).
